# Thermomechanical effect in molecular crystals: the role of halogen-bonding interactions

**DOI:** 10.1107/S2052252517014658

**Published:** 2017-10-27

**Authors:** Sudhir Mittapalli, D. Sravanakumar Perumalla, Jagadeesh Babu Nanubolu, Ashwini Nangia

**Affiliations:** a School of Chemistry, University of Hyderabad, Professor C. R. Rao Road, Gachibowli, Hyderabad 500 046, India; b Department of Inorganic and Physical Chemistry, Indian Institute of Science, Bengaluru 500 016, India; c CSIR-Indian Institute of Chemical Technology, Tarnaka, Hyderabad 500 007, India; d CSIR-National Chemical Laboratory, Dr Homi Bhabha Road, Pune 411 008, India

**Keywords:** halogen bonds, hydrogen bonds, materials science, polymorphism, salinazid, crystal engineering, mechanochemistry, molecular crystals, materials modelling

## Abstract

The design and synthesis of mechanically responsive materials is interesting because they are potential candidates to convert thermal energy into mechanical work. Reported in this paper are thermosalient effects in a series of halogen derivatives of salinazids. The chloro derivative, with higher electronegativity and a weaker inter-halogen bond strength (Cl⋯Cl) exhibits an excellent thermal response, whereas the response is weaker in the iodo derivative with stronger I⋯I halogen bonding. 3,5-Di­chloro­salinazid (Compound-A) exists in three polymorphic forms, two room-temperature polymorphs (Forms I and II) and one high-temperature modification (Form III). The transformation of Form I to Form III upon heating at 328–333 K is a reversible thermosalient transition, whereas the transformation of Form II to Form III is irreversible and non-thermosalient. 3,5-Di­bromo- (Compound-B) and 3-bromo-5-chloro- (Compound-C) salinazid are both dimorphic: the Form I to Form II transition in Compound-B is irreversible, whereas Compound-C shows a reversible thermosalient effect (362–365 K). In the case of 3,5-di­iodo­salinazid (Compound-D) and 3,5-di­fluoro­salinazid (Compound-E), no phase transitions or thermal effects were observed. The thermosalient behaviour of these halosalinazid molecular crystals is understood from the anisotropy in the cell parameters (an increase in the *a* axis and a decrease in the *b* and *c* axes upon heating) and the sudden release of accumulated strain during the phase transition. The di-halogen salinazid derivatives (chlorine to iodine) show a decrease in thermal effects with an increase in halogen-bond strength. Interestingly, Compound-B shows solid-state photochromism in its polymorphs along with the thermosalient effect, wherein Form I is cyan and Form II is light orange.

## Abstract 2

Among the halogen derivatives of salinazid, the chloro and bromo analogues show a mechanical response of jumping and breaking upon heating. Such a thermosalient response is ascribed to the sudden release of accumulated strain during the phase transition and to anisotropy in the cell parameters.

## 1. Introduction   

The development of mechanically responsive materials which respond to external stimuli such as heat, light and pressure is important because these materials are known to be self-actuating and energy-harvesting (Kim *et al.*, 2013 ▸; Morimoto & Irie, 2010 ▸; McCrone, 1965 ▸; Panda *et al.*, 2015 ▸). They have wide-ranging applications, such as artificial muscles and biomimetic and technomimetic materials (Cabane *et al.*, 2012 ▸; Ikeda *et al.*, 2007 ▸; Lehn, 2002 ▸; Mather, 2007 ▸; Rowan, 2009 ▸; Sagara & Kato, 2009 ▸; Sato, 2016 ▸; Takashima *et al.*, 2012 ▸). The design of smart materials with reversibility, stability and controlled properties is challenging (Mao *et al.*, 2016 ▸; Park *et al.*, 2016 ▸). The properties of solid-state crystalline materials depend not only on the molecular functional groups but, more significantly, on the intermolecular interactions, hydrogen bonding and molecular packing in the crystal structure. The mobility of molecules in the solid state is limited and hence when an external stimulus is applied the molecules become activated and undergo a chemical reaction or polymorphic phase change along with mechanical movement, referred to as dynamic molecular crystals, or without movement (Lieberman *et al.*, 2000 ▸; Naumov *et al.*, 2015 ▸; Swapna *et al.*, 2016 ▸; Sato, 2016 ▸). The dynamic behaviour of crystals towards applied heat (thermosalient effects; Lusi & Bernstein, 2013 ▸; Nath *et al.*, 2014 ▸; Sahoo *et al.*, 2013 ▸; Skoko *et al.*, 2010 ▸; Takeda & Akutagawa, 2016 ▸) or light energy (photosalient effect; Medishetty *et al.*, 2015 ▸) can occur with or without a change in the crystal morphology and packing of the molecules. The first reported example of the thermosalient effect was (phenylazophenyl)palladium hexafluoroacetylacetonate, an organometallic compound whose crystals were shown to jump/expand during phase transition (Etter & Siedle, 1983 ▸). Yao *et al.* (2014 ▸) reported the Ni complex [Ni^II^(en)_3_](ox) [en= ethylene­diamine; ox = oxalate] with a reversible shape change and deformation of the crystals due to a 90° rotation of the oxalate anion during phase transition and anisotropy in the unit-cell parameters. Das *et al.* (2010 ▸) reported deformation of (*S*,*S*)-octa-3,5-diyn-2,7-diol crystals due to the anisotropy in the cell parameters and a change in the stacking angle from 54 to 51°. Panda *et al.* (2016 ▸) reported positive and negative thermal expansion in thermosalient *N*′-2-propylydene-4-hydroxybenzohydrazide crystals. There have been a few reports of dynamic organic molecular crystals exhibiting jumping, curling, rolling and explosion upon external stimulus (Kim *et al.*, 2013 ▸; Koshima *et al.*, 2009 ▸; Kumar *et al.*, 2013 ▸; Baker, 2017 ▸; Uchida *et al.*, 2008 ▸).
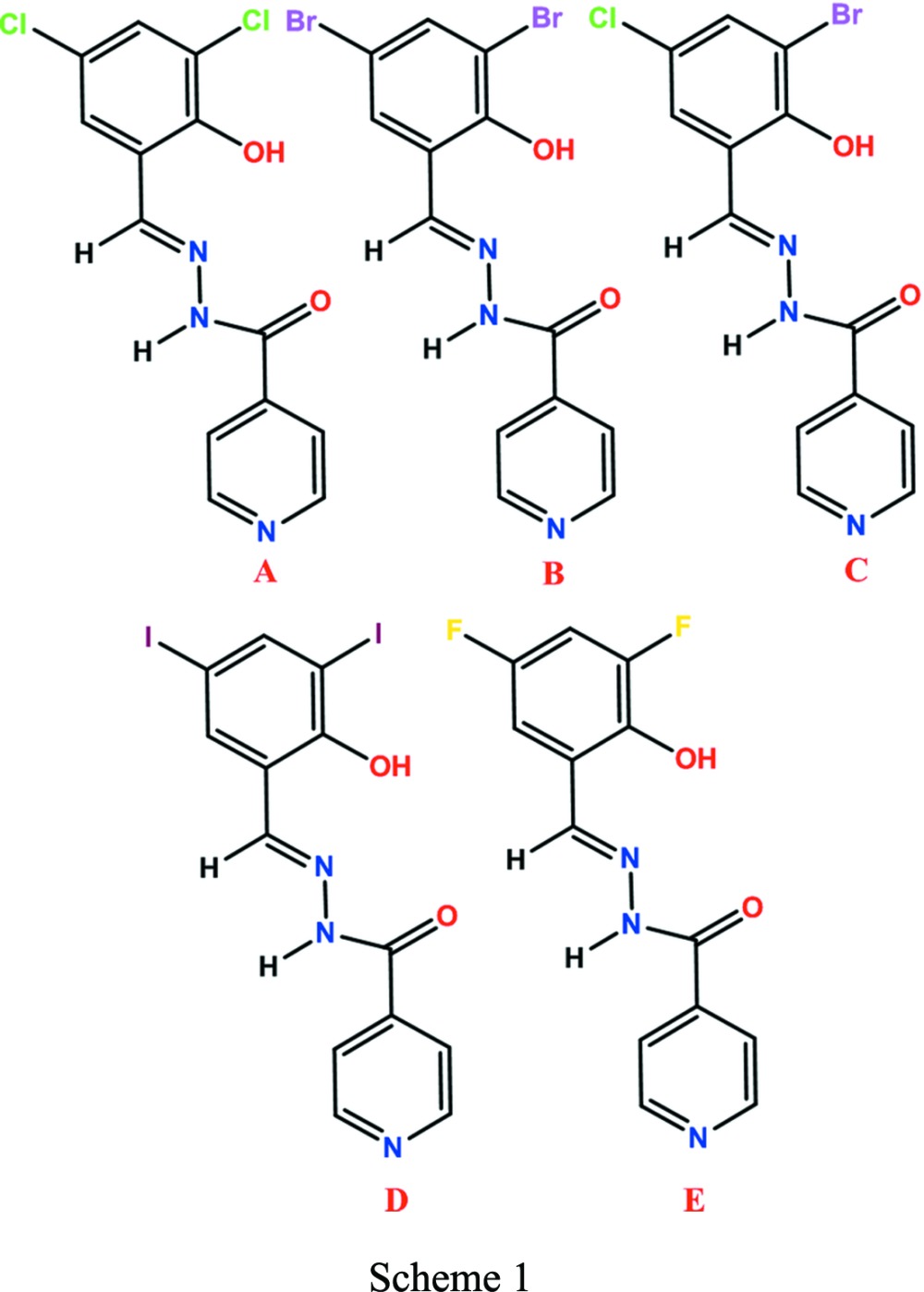


In the present investigation, a series of di-halosubstituted (Cl, Br, I and F) Schiff bases showing thermal responses and solid-state photochromism are reported. The di­chloro- (Compound-A), di­bromo- (Compound-B) and 3-bromo-5-chloro- (Compound-C) salinazid derivatives showed jumping/breaking/cracking of the crystals during phase transformation. Phase transformation with a change in *Z*′ from 1 to 2 was observed for Compound-B, with significant changes in the molecular conformation (dihedral angles), hydrogen bonding and crystal packing. Crystals of Compound-D and E (diiodo- and difluoro-salinazid) did not show any type of thermal response on heating. Chemical diagrams are shown in Scheme 1, and the 2-hy­droxy­benzyl­idene isonicotinohydrazide skeleton is referred to as salinazid.

## 2. Experimental   

For the preparation of di­chloro­salinazid Compound-A, (*E*)-*N*′-(3,5-di­chloro-2-hydroxybenzylidene)isonicotinohydrazide, the conventional reaction with azeotropic removal of water (Safin *et al.*, 2014 ▸) was followed. Isonicotinohydrazide (1 mmol, 137 mg) was dissolved in anisole (10 ml) in a 100 ml round-bottomed flask fitted with a Dean–Stark set up. 3,5-Dichlorosalicylaldehyde (1 mmol, 191 mg) was added to the solution. The reaction mixture turned light yellow (after 10 min) and it was refluxed for 4 h. The solution was concentrated by removing the solvent and was then washed with *n*-hexane 3–4 times to remove unwanted by-products. Light-cyan crystals of dichloro Compound-A were obtained in 80% yield. The same procedure was followed for the synthesis of Compounds-B, C, D and E, and their structures were confirmed by ^1^H NMR and ^13^C NMR (Figs. S1*a*–S1*h* in the supporting information) and finally by single-crystal X-ray diffraction.

### 2.1. Compound-A   

^1^H NMR (400 MHz, *d*_6_-DMSO, 298 K, δ in p.p.m., Fig. S1*a*): 12.73 (*s*, 1H), 12.30 (*s*, 1H), 8.82 (*d*, *J* = 2.4 Hz, 2H), 7.86 (*d*, *J* = 2.8 Hz, 2H), 7.73 (*d*, *J* = 2.56 Hz, 1H), 7.66 (*d*, *J* = 2.48 Hz, 1H), 7.45 (*br*, 1H).

^13^C NMR (400 MHz, *d*_6_-DMSO, 298 K, δ in p.p.m., Fig. S1*b*): 166.7, 157.4, 155.6, 153.2, 153.1, 144.4, 135.6, 133.5, 128.2, 126.8 and 125.8.

FT–IR (KBr, cm^−1^, Fig. S2*a*): 3193.3 (*br*, OH), 1677.8 (*m*, C=O), 1612.7 (*m*, C=N), 1555.6 (*w*), 1498.6 (*w*), 1451.7 (*m*), 1434.1 (*w*), 1349.4 (*w*), 1180 (*m*).

### 2.2. Compound-B   

^1^H NMR (400 MHz, *d*_6_-DMSO, 298 K, δ in p.p.m., Fig. S1*c*): 12.78 (*s*, 1H), 12.53 (*s*, 1H), 8.82 (*d*, *J* = 5.6 Hz, 2H), 8.56 (*s*, 1H), 7.85 (*t*, *J* = 2.8 Hz, 4H).

^13^C NMR (400 MHz, *d*_6_-DMSO, 298 K, δ in p.p.m., Fig. S1*d*): 162.1, 154.1, 150.9, 148.5, 139.7, 136.3, 132.6, 122.0, 121.3, 111.8 and 111.7.

FT–IR (KBr, cm^−1^, Fig. S2*b*): 3149.7 (*br*, OH), 1685.3 (*m*, C=O), 1593.9 (*m*, C=N), 1549.5 (*w*), 1441.8 (*w*), 1370.3 (*m*), 1340.1 (*w*), 1269.1 (*w*), 1218.7 (*m*).

### 2.3. Compound-C   

^1^H NMR (400 MHz, *d*_6_-DMSO, 298 K, δ in p.p.m., Fig. S1*e*): 12.76 (*s*, 1H), 12.50 (*s*, 1H), 8.83 (*d*, *J* = 6.0 Hz, 2H), 8.58 (*s*, 1H), 7.87 (*d*, *J* = 6 Hz, 2H), 7.78 (*d*, *J* = 3.2 Hz, 2H).

^13^C NMR (400 MHz, *d*_6_-DMSO, 298 K, δ in p.p.m., Fig. S1*f*): 166.8, 158.5, 155.6, 153.3, 144.4, 138.5, 134.5, 128.6, 126.7, 125.4 and 116.1.

### 2.4. Compound-D   

^1^H NMR (400 MHz, *d*_6_-DMSO, 298 K, δ in p.p.m., Fig. S2*g*): 12.77 (*m*, 2H), 8.83 (*m*, 2H), 8.47 (*s*, 1H), 8.08 (*d*, *J* = 1.2 Hz, 1H), 7.95 (*s*, 1H), 7.85 (*t*, *J* = 4.8 Hz, 2H).

### 2.5. Compound-E   

^1^H NMR (400 MHz, *d*_6_-DMSO, 298 K, δ in p.p.m., Fig. S2*h*): 12.46 (*s*, 1H), 11.12 (*s*, 1H), 8.81 (*d*, *J* = 4.8 Hz, 2H), 8.69 (*s*, 1H), 7.85 (*d*, *J* = 4.8 Hz, 2H), 7.39 (*m*, 2H).

### 2.6. X-ray crystallography   

X-ray reflections for Compounds-A (Form I and Form II), -B (Form I and Form II), -C (Form I), -D and -E were collected on a Bruker D8 Quest CCD diffractometer equipped with a graphite monochromator and an Mo *K*α fine-focus sealed tube (λ = 0.71073 Å) at 298 K. High-temperature data for Compound-A Form III and Compound-C Form II were collected on a Bruker D8 Venture diffractometer at 338 and 368 K, respectively. Data reduction was performed using Bruker *APEX2* software. Intensities were corrected for absorption using *SADABS* (Sheldrick, 1997*a* ▸) and the structures were solved and refined using *SHELX97* (*SMART 2000* ▸; Sheldrick, 1997*b* ▸). All non-H atoms were refined anisotropically. H atoms on hetero atoms were located from difference electron-density maps and all C-bound H atoms were fixed geometrically. *ORTEP* plots are shown in Fig. S3 in the supporting information. Hydrogen-bond geometries were determined using *PLATON* (Spek, 2002 ▸). *X-Seed* (Barbour, 1999 ▸) was used to prepare packing diagrams. The crystal structures have been deposited with the Cambridge Structural Database (CCDC Numbers 1548278–1548285). Compound-A Form I is reported in the CCDC as No. 824932.

### 2.7. Powder X-ray diffraction (PXRD)   

Powder X-ray diffraction was recorded on Bruker D8 Advance diffractometer (Bruker AXS, Karlsruhe, Germany) using Cu *K*α X-radiation (λ = 1.5406 Å) at 40 kV and 30 mA power. X-ray diffraction patterns were collected over the 2θ range 5–40° at a scan rate of 1° min^−1^.

### 2.8. Infrared spectroscopy (IR)   

A Nicolet 6700 FT–IR spectrometer with an NXR FT–Raman Module was used to record IR spectra. IR spectra were recorded on samples dispersed in KBr pellets.

### 2.9. Differential scanning calorimetry (DSC)   

DSC was performed on a Mettler Toledo DSC 822e module. Samples were placed in crimped but vented aluminium sample pans. The typical sample size was 3–5 mg. The temperature range was 303–573 K at 20 K min^−1^. Samples were purged by a stream of nitro­gen flowing at 60 ml min^−1^.

### 2.10. Thermogravimetric analysis (TGA)   

TGA was carried out using a Mettler Toledo TGASDTA 851e operating with *STAR^e^* software to detect solvates and thermal degradation. Accurately weighed (5–15 mg) samples were loaded in alumina crucibles and heated at a rate of 20 K min^−1^ over a temperature range of 303 to 573 K under a nitro­gen purge of 60 ml min^−1^.

### 2.11. Hot-stage microscopy (HSM)   

HSM was performed on a Wagner & Munz Polytherm A Hot Stage and Heiztisch microscope. A Moticam 1000 (1.3 MP) camera supported by software *Motic Image Plus 2.0ML* was used to record images and videos (Fig. S4 in the supporting information).

### 2.12. Computations   

The stabilization energies of all complexes were computed using M062X/6-31g(d,p) (SDD for Br and I) (Zhao & Truhlar, 2008 ▸) in the *GAUSSIAN09* package (Frisch *et al.*, 2009 ▸). All energies are BSSE-corrected using the counterpoise method.

## 3. Results and discussion   

**Figure 1:**
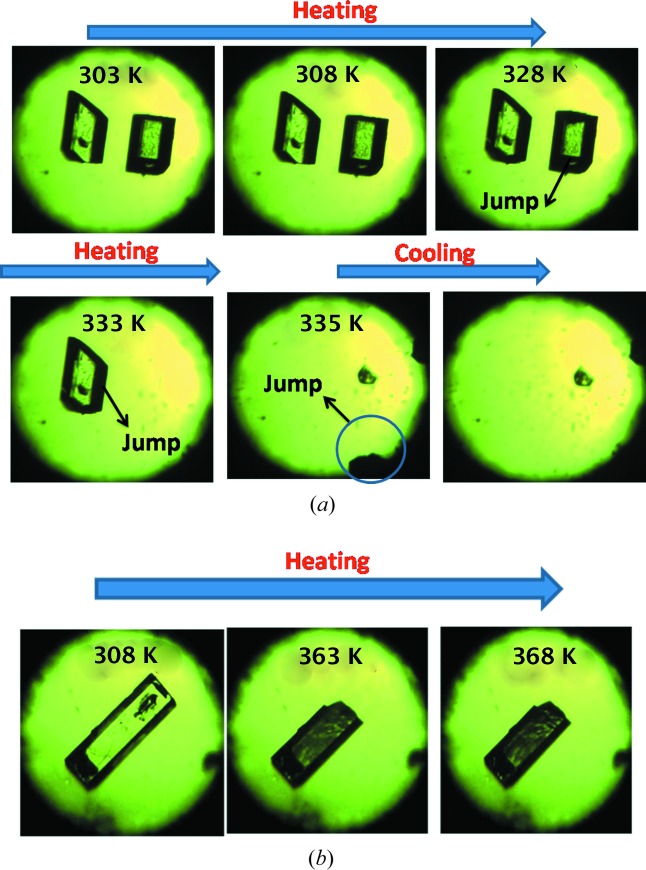
(*a*) HSM images of Compound-A Form I crystals during heating. (*b*) HSM images of Compound-C Form I crystals, and separation of debris during heating.

The dichloro Compound-A was crystallized from methanol multiple times and finally provided diffraction-quality single crystals of Form I. Form II crystals were obtained from a methanol slurry after 1 d. The high-temperature polymorph Form III was isolated by heating and it is stable at high temperature (above 330 K, HT polymorph). Crystallization experiments on compounds-B, -C, -D and -E provided dimorphs of B and C, while D and E each gave a single-crystal form. Scheme 2 shows the thermal-induced and crystallization transformation for polymorphs of Compound-A and Scheme 3 shows thermosalient (TS) effects on Compound-C. Preliminary observations of crystal changes were visualized on a hot-stage microscope (HSM). Upon heating Form I crystals of A to about 328–338 K, a few crystals were seen to fly off suddenly from the hot stage to a few centimetres in height (1–2 cm, Fig. 1 ▸ *a*) and moved outside the camera zone, while other bits were fragmented into small pieces during heating. Form II crystals of A did not exhibit any thermal response. Crystals of B showed cracking upon heating (Fig. S4*a* in the supporting information) and Compound-C led to rapid breaking and separation of debris (Fig. 1 ▸ *b*) at 363–368 K. Crystals of compounds-D and E were unresponsive to thermal stress.
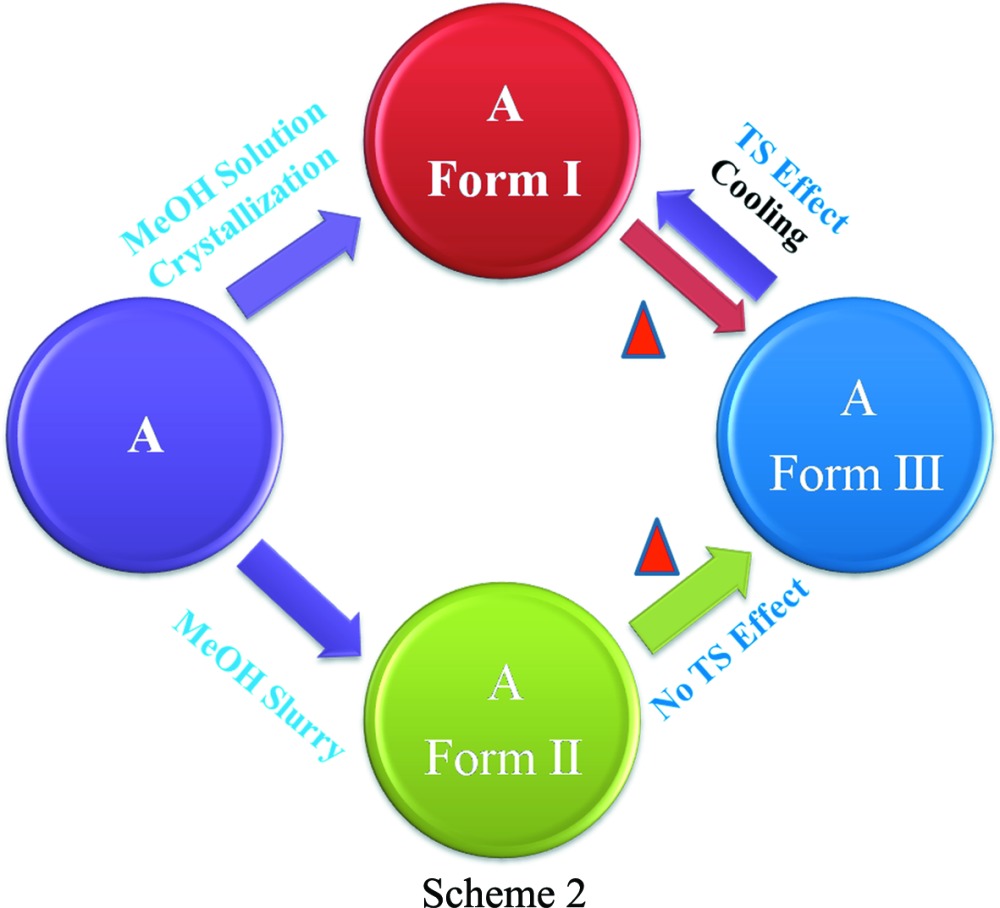
 
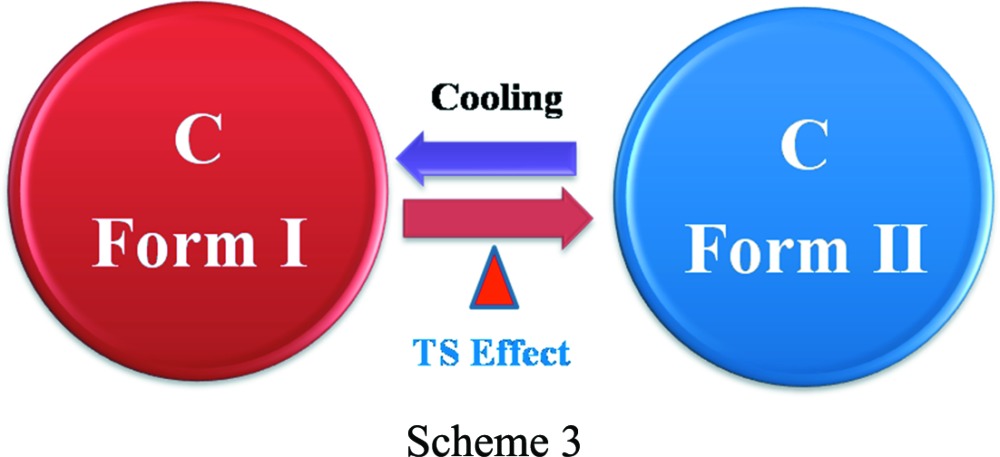


### 3.1. X-ray crystal structures   

**Figure 2:**
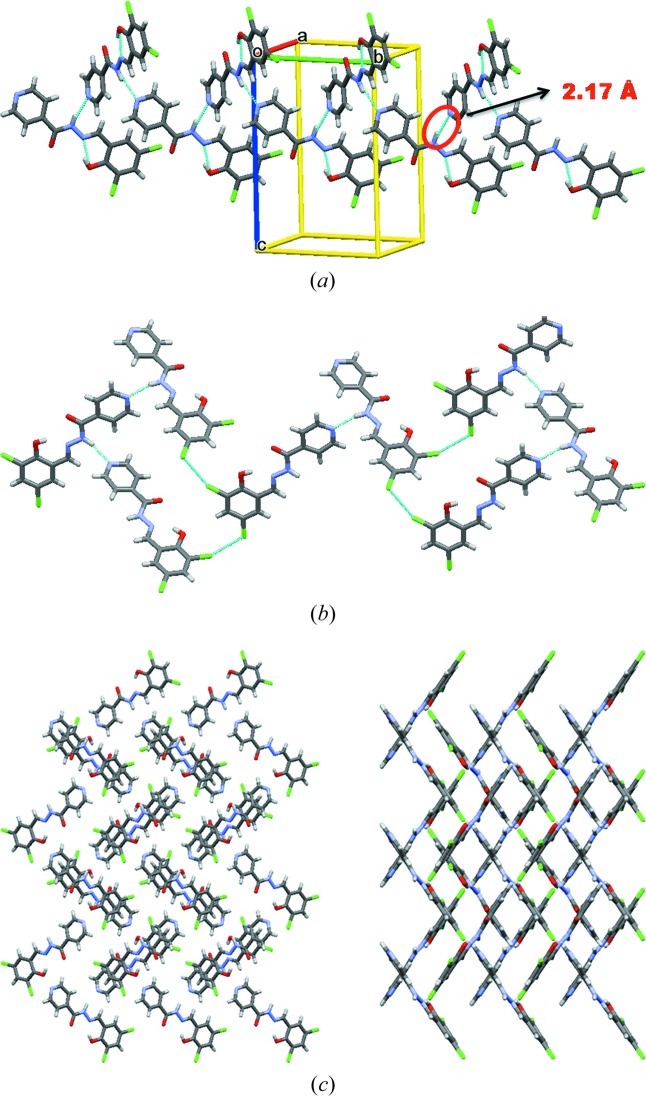
(*a*) The one-dimensional chain of Form I of Compound-A propagating through the N—H⋯N (pyridine) synthon. (*b*) Type II halogen bonding. (*c*) Packing of molecules along the *a* and *c* axes (Form I).

**Figure 3:**
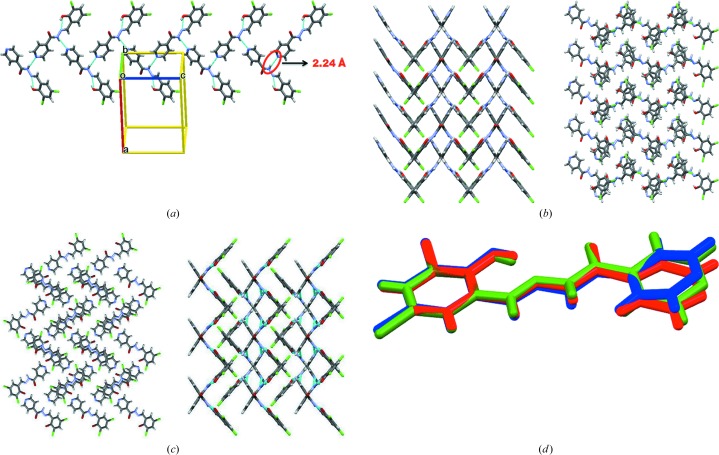
(*a*) The one-dimensional chain of Form II of Compound-A propagating through the N—H⋯N (pyridine) synthon. (*b*) Packing of molecules along the *a* and *b* axes (Form II). (*c*) Packing of molecules along the *a* and *c* axes (Form III). (*d*) Overlay of the Form I (green), Form II (blue) and Form III (red, high-temperature polymorph) crystal structures (Compound-A).

The X-ray crystal structures of Compound-A Form I and III were solved in the monoclinic space group *P*2_1_/*c*. The hydrazine N—H bonds to the pyridine *N*-acceptor [N2—H2*B*⋯N3 = 2.17 (1) Å and 156 (4)° for Form I, and 2.23 (1) Å and 158.7 (1)° for Form III; Fig. 2 ▸ *a*] to form a chain along the *b* axis. Such chains extend through type II halogen bonds [Form I: C—Cl⋯Cl—C, θ_1_ = 155.44 (5)°, θ_2_ = 95.96 (5)°; Form III: θ_1_ = 155.5 (4)°, θ_2_ = 95.2 (3)°; Fig. 2 ▸ *b*] (Metrangolo & Resnati, 2014 ▸; Mukherjee & Desiraju, 2014 ▸). An intramolecular hydrogen bond is present between the imine N atom and the phenolic H atom [O2—H2*A*⋯N1 = 1.86(2) Å and 145° and 1.88 (1) Å and 144.1 (4)° for Forms I and III, respectively, forming an *S*(6) ring motif. In the case of Form II, crystals were obtained by fast evaporation of solvent from the solution and the structure was solved and refined in orthorhombic space group *Pca*2_1_ with one molecule in the asymmetric unit. Similar to Form I, Form II also has the same type of one-dimensional chains (Fig. 3 ▸ *a*) and the chain grows parallel to the *c* axis. The one-dimensional chains are extended by type II [C—Cl⋯Cl—C, θ_1_ = 151.2 (1)°, θ_2_ = 100.21 (8)°] halogen-bonding interactions (Saha *et al.*, 2006 ▸). Similar to Forms I and III, Form II also reveals the same types of hydrogen- and halogen-bonding interactions except for changes in the halogen/hydrogen-bonding distances (Table 1 ▸).

**Figure 4:**
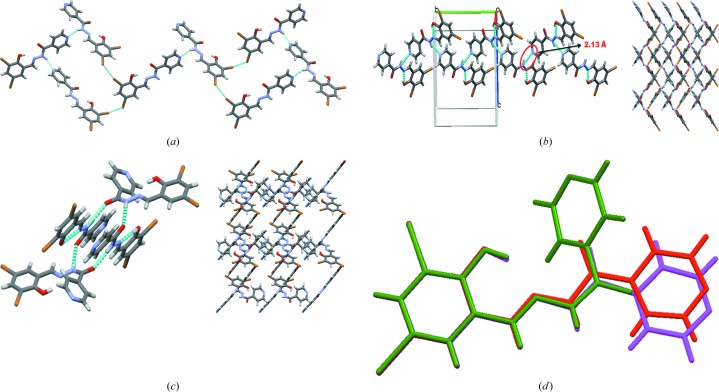
(*a*) Type II halogen-bonding interactions of Compound-B. (*b*) The one-dimensional chain propagating through the N—H⋯N (pyridine) synthon (Form I) and packing of molecules along the *c* axis. (*c*) The tetrameric ring motif (Form II) and packing diagram (along the *c* axis). (*d*) Overlay of Form I (red) and Form II (green and magenta) of Compound-B.

Compound-B Form I crystallized from methanol solvent and was solved in the monoclinic crystal system, space group *P*2_1_/*c*, with one molecule in the asymmetric unit. Similar to Form I of the dichloro derivative, the hydrazine H atom interacts with the pyridine N atom through an N2—H2*B*⋯N3 hydrogen bond [2.13 (2) Å and 152.4 (1)°], forming a one-dimensional chain (Fig. 4 ▸ *a*). These chains grow parallel to the *b* axis and are extended by type II halogen bonds [C—Br⋯Br—C, θ_1_ = 166.0 (1)°, θ_2_ = 94.4 (1)° Fig. 4 ▸ *b*]. Form II was obtained by heating Form I crystals. It crystallized in the triclinic space group 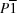 with two molecules in the asymmetric unit. A tetrameric ring motif is formed through N2—H2*B*⋯O3 [2.15 (1) Å and 164.2 (1)°] and N5—H5*B*⋯O1 [2.11 (1) Å and 168.9 (1)°; Fig. 4 ▸ *c*] hydrogen-bonding interactions, forming an 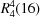 motif. The tetrameric rings are extended by type I halogen bonds [C—Br⋯Br—C, θ_1_ = θ_2_ = 142.6 (2)°]. There are significant differences in the dihedral angles between the two rings before [Form I, ring atom numbers C1–C6 and C9–C13/N3 15.55 (3)°] and after phase transformation [Form II, ring atom numbers C14–C19 and C22–C26/N6 41.36(3)°, and ring atom numbers C1–C6 and C9–C13/N3 4.47 (4)°; Table 2 ▸].

**Figure 5:**
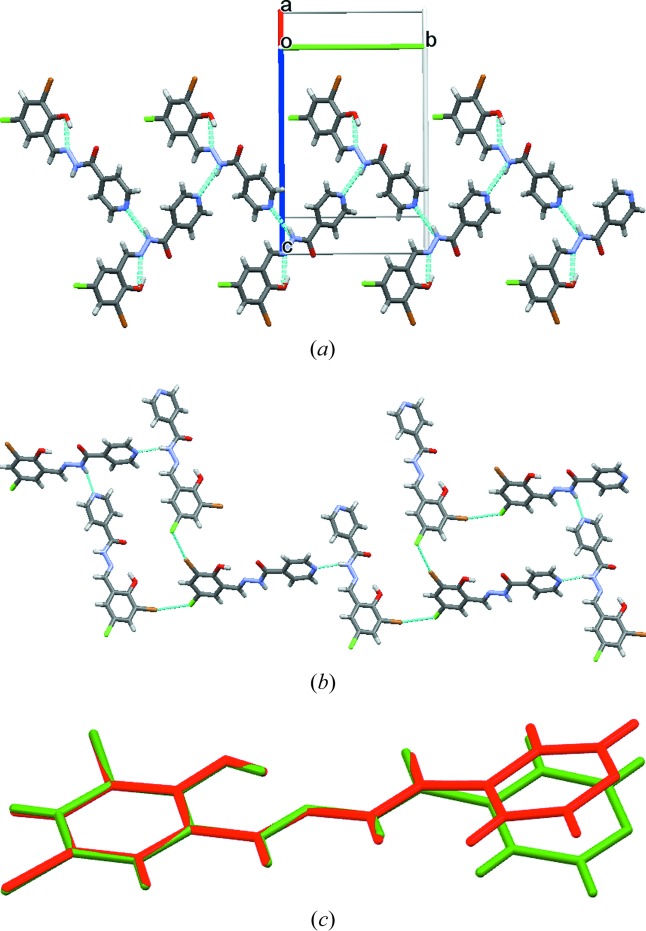
(*a*) The one-dimensional chain propagating through the N—H⋯N (amino-pyridine) synthon (Form I, Compound-C). (*b*) Cl⋯Br type II halogen-bonding interactions. (*c*) Overlay of Form I (green) and Form II (red) molecules.

Similar to the above two compounds, Compound-C (Forms I and II) also has the same types of hydrogen- and halogen-bonding interactions: N2—H2*B*⋯N3 [2.16 (3) Å and 156 (2)°, and 2.22 (3) Å and 156 (3)°] in a one-dimensional chain (Fig. 5 ▸ *a*) and type II [Form I, C—Cl⋯Br—C, θ_1_ = 167.48 (8)°, θ_2_ = 98.49 (9)°; Form II, C—Cl⋯Br—C, θ_1_ = 163.1 (1)°, θ_2_ = 97.9 (1)°; Fig. 5 ▸ *b*] halogen bonds between the chains.

**Figure 6:**
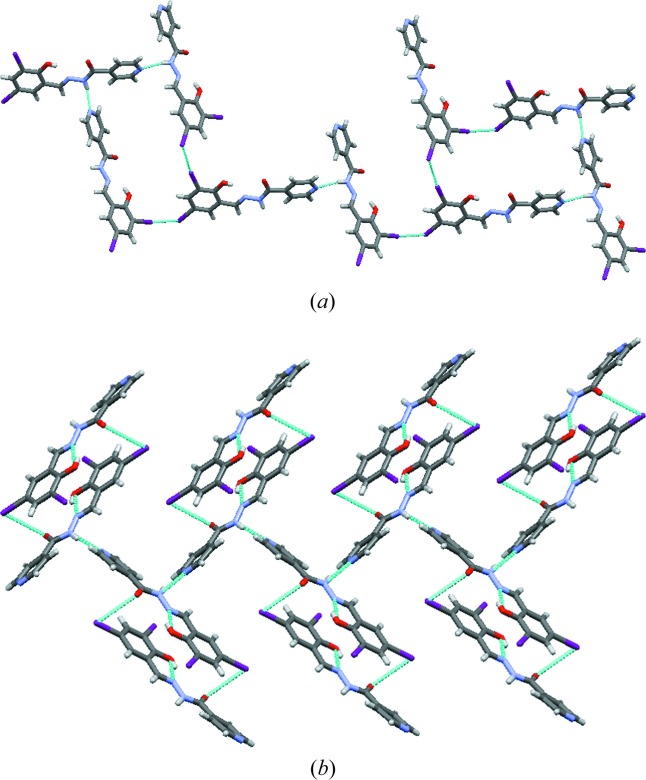
(*a*) Type II (I⋯I) halogen bonding of Compound-D. (*b*) The dimers extended *via* N—H⋯N hydrogen bonds (Compound-D).

In the crystal structure of Compound-D, dimers are formed through C—I⋯O halogen-bonding interactions and these dimers are extended *via* type II halogen bonds [C—I⋯I—C, 3.856 Å and θ_1_ = 170.3 (1)°, θ_2_ = 91.95 (9)°; Fig. 6 ▸ *a*] and N2—H2*B*⋯N3 [2.15 (5) Å and 146 (5)°; Fig. 6 ▸ *b*) hydrogen-bonding interactions.

Similar to the above structures, in Compound-E the same type of N2—H2*B*⋯N3 hydrogen bonds are present (Fig. S5 in the supporting information) but halogen bonding is absent for the fluoro group, which does not generally engage in intermolecular interactions (Dikundwar *et al.*, 2014 ▸; Chopra & Row, 2011 ▸).

### 3.2. VT-PXRD and thermal analysis   

**Figure 7:**
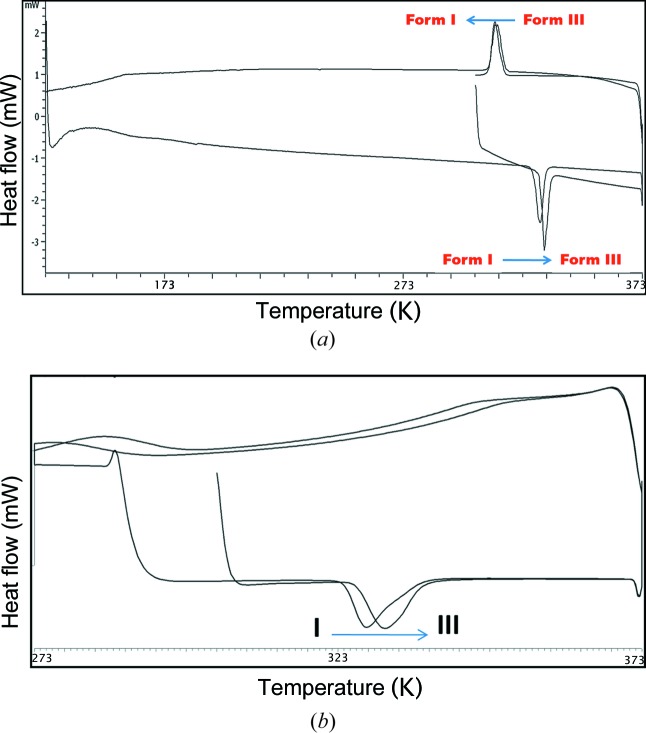
(*a*) DSC thermogram of Compound-A Form I crystals (heat–cool–reheat). (*b*) DSC thermogram of Compound-A Form I ground material (heat–cool–reheat).

**Figure 8:**
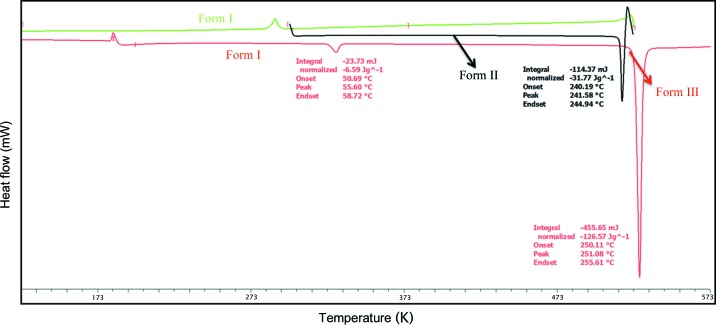
DSC thermogram of Compound-A Form II (heat–cool–reheat).

**Figure 9:**
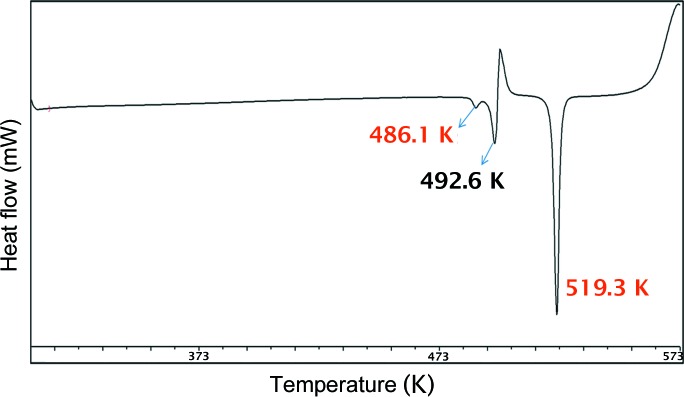
DSC thermogram of Compound-B Form I.

In order to understand the events observed on the hot-stage microscope, both DSC and variable-temperature PXRD were performed on all the materials. Heating Compound-A, Form I showed an endotherm at 330.4 K, indicating the transformation of Form I to Form III (Nauha & Bernstein, 2014 ▸). Cooling the same material exhibited an exotherm at 308–318 K, indicating Form III → Form I conversion. Reheating the same material showed an endotherm at 328.4 K (Form I → Form III) followed by melting at 523.9 K (Fig. 7 ▸ *a*, and Fig. S6*a* in the supporting information). These observations suggest that Forms I and III are enantiotropic polymorphs and that Form III is stable after the phase transition (high-temperature polymorph, VT-PXRD plots in Fig. 6 ▸ *b*). In the case of Form II (Compound-A) an endotherm was observed at 513.3 K after Form II converted into Form III. On cooling the same solid, Form III transformed into Form I and showed an exotherm at 278–293 K. On reheating the same material, an endotherm was observed at 323.8 K followed by melting at 523.3 K (Fig. 8 ▸). Apart from heating, competitive slurry experiments (in solution medium, methanol as solvent) suggest that Form II is more stable than Form I. Heating Compound-B showed a small endotherm at 486.1 K and a second endotherm at 492.6 K, which indicates the transformation of Form I into Form II, followed by melting at 519.3 K (Fig. 9 ▸). This means that Forms I and II are enantiotropically related.

**Figure 10:**
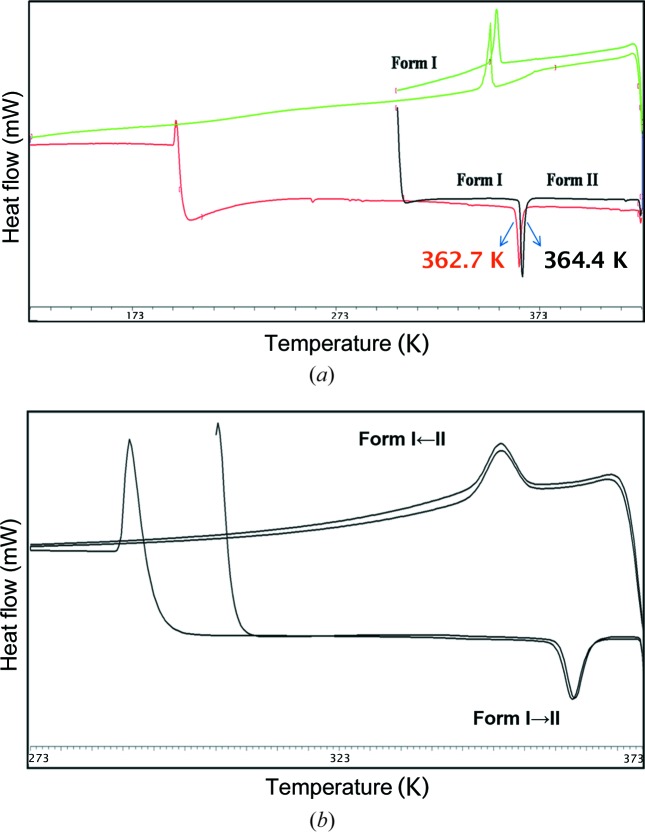
(*a*) DSC thermogram of Compound-C Form I (heat–cool–reheat). (*b*) DSC thermogram of Compound-C Form I ground material (heat–cool–reheat).

In case of Compound-C, an endotherm was observed at 364.4 K for the conversion of Form I to Form II. Cooling the same material showed an exotherm at 343–353 K for the conversion of Form II to Form I, and on reheating of the material an endotherm was observed at 362.7 K followed by melting at 528.5 K (Fig. 10 ▸ *a*, and Fig. S7*a* in the supporting information). The transformation of Form I and Form II is reversible and Form II is stable at high temperature (above 393 K, Fig. S7*b*). Compounds-D and E melted at 518 and 560 K, respectively, without any phase transformation, indicating the absence of polymorphism in these two compounds (Figs. S8 and S9). DSC of the ground material (Figs. 7 ▸ *b* and 10 ▸ *b*) showed the same type of reversible phase transitions, wherein the intensity of the peaks was less than those of the single-crystal material thermal profile, indicating that single crystals can withstand the pressure which is accumulated during heating and the release of strain compared with the powder solids. The crystalline materials undergo phase transitions in a very short time (microseconds) and this sudden change is the probable reason for the thermosalient effect. All the observed changes were verified by HSM, DSC and VT-PXRD (Figs. S4, S6, S7, S8, S9 in the supporting information). All experiments were performed multiple times to confirm reproducibility.

### 3.3. Powder X-ray analysis   

Powder XRD is a nondestructive technique and most useful for the identification of different powder materials. Signature lines for Compound-A (Form I) were observed at 2θ 9.9, 10.9, 14.0, 14.7; Form II at 10.6, 12.0, 14.1, 16.7 and Form III at 10.3, 13.3, 14.3, 17.6; Compound-B Form I at 9.8, 11.3, 12.6, 15.0; Form II at 8.4, 9.0, 13.1, 18.0; Compound-C Form I at 9.9, 10.7, 13.9, 15.1; Form II at 10.2, 14.1, 17.8, 19.0 (Fig. S10).

### 3.4. Thermomechanical effect and role of halogen bonding   

**Figure 11:**
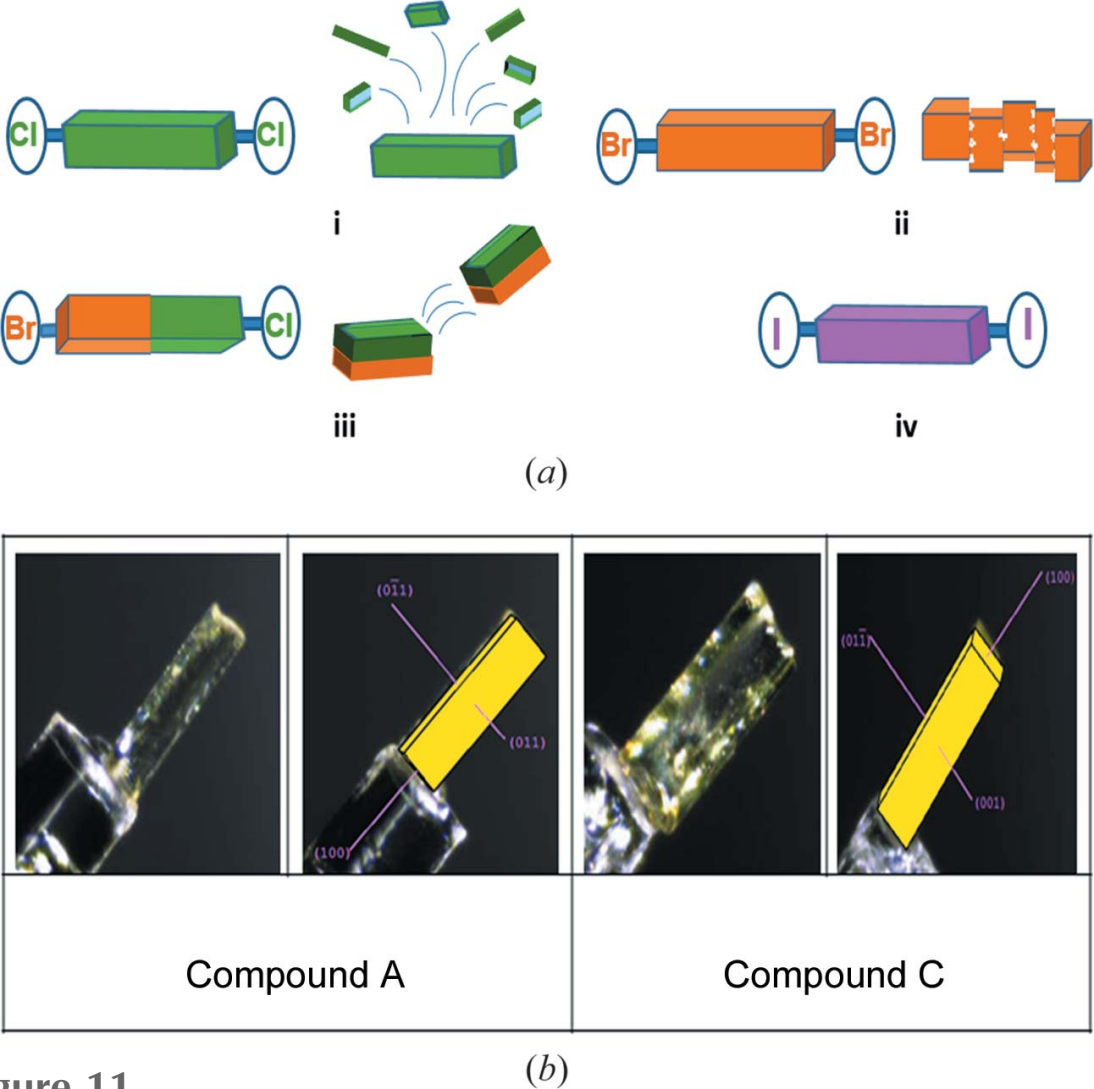
(*a*) Thermosalient effects of crystals, such as (i) jumping/explosion, (ii) cracking and (iii) rapid breaking of a crystal into two pieces during the phase transition. (iv) The iodine derivative crystal is intact upon heating. (*b*) Morphology and faces of Form I crystals of Compounds-A and C.

**Figure 12:**
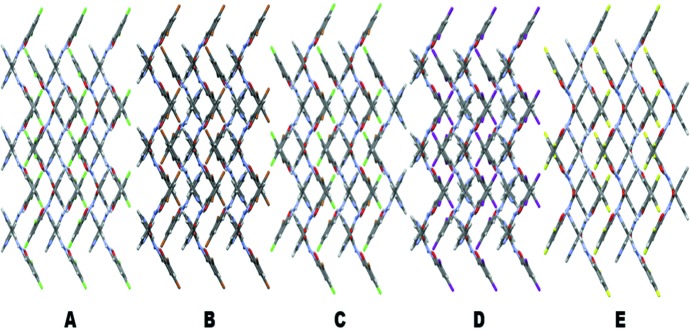
Packing of molecules in the halogen derivatives A to E to show the similarity.

**Figure 13:**
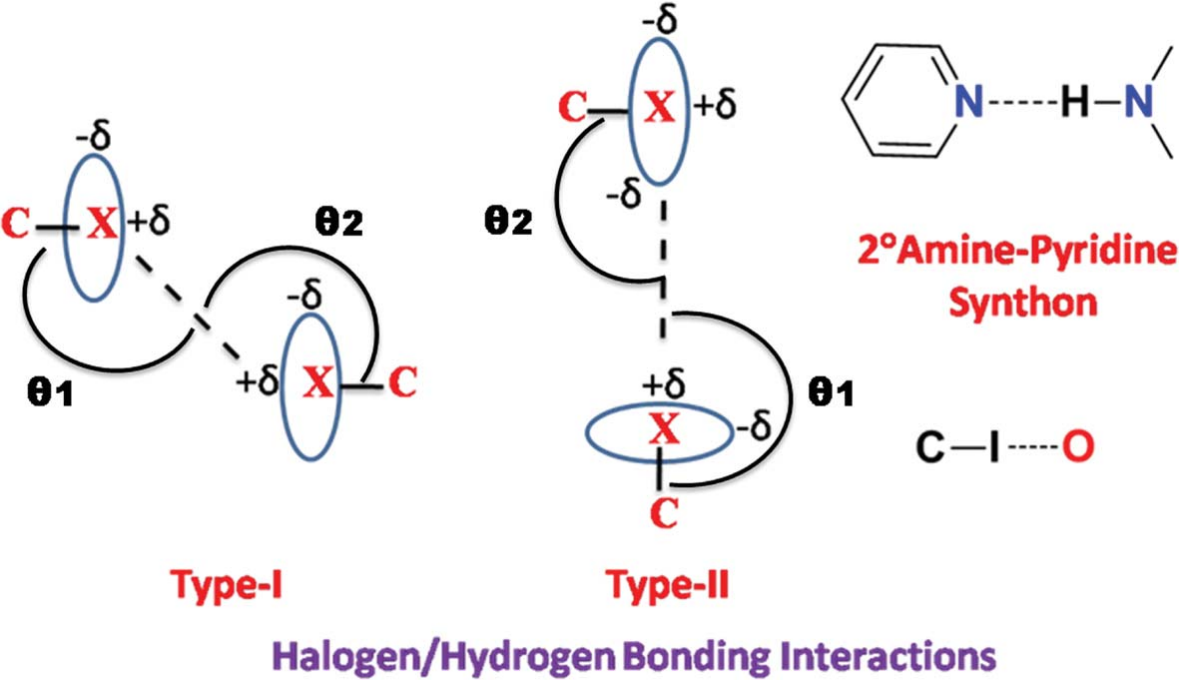
Halogen-bonding interactions and hydrogen-bonding synthons in the present study.

Different thermal events (jumping/separation of debris, and cracking; Fig. 11 ▸ *a*) were observed in halogen derivatives such as Compounds-A, -B, and -C even though they are isomorphous (Table 3 ▸) and three-dimensionally isostructural (*XPac* analysis; Fig. S11; Gelbrich *et al.*, 2012 ▸) and have the same type of hydrogen/halogen-bonding interactions and similar packing (Fig. 12 ▸), except for the change of halogen atom. During heating of Compound-A, jumping/explosion of crystals later converted into fine powder, Compounds-B and C showed breaking and cracking of the crystal into pieces during the phase transition, and no effect was observed in Compounds-D and E. In the present study, all compounds except B (Form II) contain type II halogen-bonding interactions, *i.e.* those with an attractive δ^+^⋯δ^–^ polarization (Metrangolo & Resnati, 2014 ▸; Mukherjee & Desiraju, 2014 ▸). Type II interactions are strong and attractive in nature (Bui *et al.*, 2009 ▸). Due to the anisotropic distribution of electrons on the heavier halogen atoms, a partial positive charge develops opposite to the sigma bond (σ hole) and a partial negative charge in the equatorial region, leading to an attractive δ^+^⋯δ^–^ interaction (Fig. 13 ▸). The exception is the fluorine atom (Thalladi *et al.*, 1998 ▸) which is hard, highly electronegative and non-polarizable. On moving from chlorine to iodine, the polar flattening of the halogen atom increases due to the decrease in electronegativity and the increase in polarizability, thereby resulting in a stronger type II inter-halogen bond (Desiraju & Parthasarathy, 1989 ▸). The electrostatic nature of type II halogen bonding (Metrangolo & Resnati, 2014 ▸; Mukherjee & Desiraju, 2014 ▸) means that crystal structures containing these interactions are more responsive to temperature changes (thermosalient effects). Saraswatula & Saha (2014 ▸) reported that thermal expansion of inter-halogen distances is higher for lower halogens (chlorine) and a decrease in expansion was observed with an increase in the halogen-bond strength. So the behaviour of heavier halogen atom-containing species is different from that of the lower halogens. Fluorine is an exception to showing halogen bonding due to its high electronegativity. In effect, the stronger halogen-bonded structures (Table 4 ▸) with Br and I atoms make them less responsive to temperature and mechanical stress because the halogen bond is too strong for the heavier halogens to show structural dynamics and mechanical property effects. The decreasing order of thermal response for inter-halogen interactions is Cl⋯Cl > Cl⋯Br > Br⋯Br > I⋯I, indicating the importance of weaker Cl⋯Cl interactions in exhibiting a mechanical response of molecular crystals and temperature effects.

**Figure 14:**
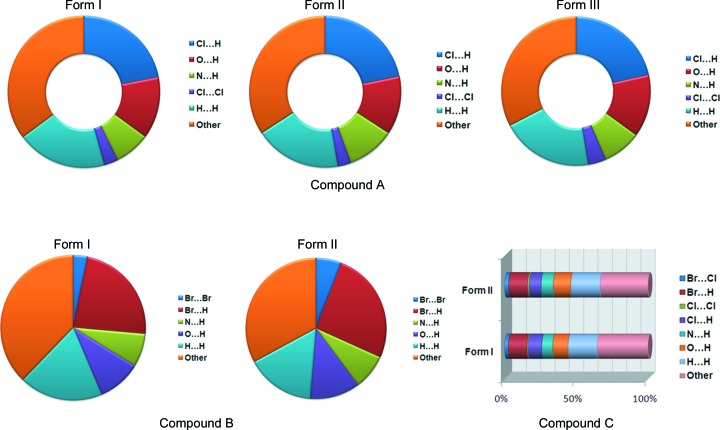
Hirshfeld surface analysis for polymorphs of Compounds-A, -B and -C, showing the contributions of the different hydrogen- and halogen-bonding interactions.

Heating the crystals of Compound-A Form I in the (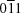)/(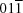) (Fig. 11 ▸ *b*) faces resulted in a thermal response of the crystals, some of which were seen to fly off the hot stage while a few others exploded into pieces at 330 K. They converted into metastable Form III, with an increase in cell parameters along the *a* axis (7.7%) and a decrease in the *b* and *c* axes (4.5 and 0.8%, respectively) and an increase in volume (of 2.1%, Table 5 ▸). During cooling the crystals moved without any disintegration. Heat–cool cycles were conducted 3–4 times, after which the crystals exhibiting a thermal response turned to powder. Similarly, Form II also converted into Form III at 513 K without any thermal response. Comparison of the two crystal structures (Forms I and II) showed changes in the hydrogen-bond distances and packing of the molecules. In a Hirshfeld analysis (Fig. 14 ▸, and Fig. S12), Form II has a greater contribution from Cl⋯H, O⋯H, N⋯H and Cl⋯Cl contacts (47.3%) compared with Form I (45.7%), and the halogen-bond distances are shorter (stronger bonding) in Form II compared with Form I (Tables 1 ▸, 4 ▸), thus supporting the non-thermosalient transformation of Form II to Form III.

On heating crystals of Compound-B Form I on the (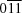)/(011) faces (Fig. S13), Form I transformed to Form II with cracking, with an increase in *Z*′ from 1 to 2 and a lengthening of the *a* axis (18.7%) and a decrease in the *b* and *c* axes (1.3 and 11.3%, respectively), but with a negligible increase in the overall cell volume (1.2%).

Rapid breaking of the crystals was observed during heating of Compound-C Form I crystals on the (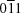)/(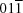) faces (Fig. 11 ▸ *b*), with an expansion on the *a* axis and contraction along the *b* and *c* axes.

To understand the thermal response of these molecular crystals, the room-temperature and high-temperature polymorphic structures of Compounds-A, -B and -C were compared. The integrity of the hydrogen-bonding interactions is retained in the structures of Compounds-A (Form I → Form III) and C (Form I → Form II) without any significant changes in dihedral angles (Table 2 ▸), hydrogen- and halogen-bond distances or packing of the molecules, before and after the phase transition (Table 1 ▸). However, in the case of Compound-A Form II → Form III and Compound-B Form I → Form II, the transition occurs with significant changes in hydrogen- and halogen-bonding interactions and in the packing of the molecules. A probable reason for the thermal response is ascribed to the rapid release of accumulated strain during the phase transition and anisotropy in the structural parameters.

### 3.5. Photochromism   

Photochromism (Safin *et al.*, 2014 ▸; Cohen *et al.*, 1964 ▸; Senier & Shepheard, 1909 ▸; Zbačnik & Kaitner, 2014 ▸) of both organic and inorganic materials is defined as the reversible colour change of a material on exposure to electromagnetic radiation such as UV, visible light or IR. The sensitivity of such materials towards light makes them potential candidates in reusable information storage media, data displays, chemical switches for computers *etc*. (Gust *et al.*, 2012 ▸; Kawata & Kawata, 2000 ▸). The salinazid derivatives presented in this study show thermochromic and photochromic behaviour in the solid state.

**Figure 15:**
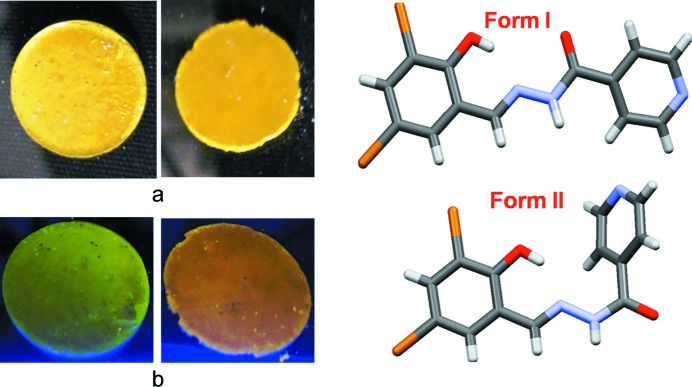
(Left) Compound-B Form I and Form II (*a*) under normal light and (*b*) under UV at 365 nm. (Right) The variation in the conformation of the molecule.

The accepted mechanism for photochromism is the transfer of a proton to the N atom (*cis*-keto) followed by isomerization of *cis*-keto to *trans*-keto isomers (Scheme 4) (Hadjoudis & Mavridis, 2004 ▸). The dihedral angle between two phenyl rings is the predictor for photochromism. If the angle is >30° then the compounds are photochromic, if the angle is 20–30° the compounds are either photochromic or non-photochromic, and if the angle is <20° then the compounds are non-photochromic (Carletta *et al.*, 2016 ▸). The polymorphs of Compound-B show photochromism in the solid state during exposure to UV light (365 nm). The dihedral angles in Form I and Form II are 15° and 42°, respectively, and the former crystal is cyan in colour and the latter is light orange (Fig. 15 ▸).
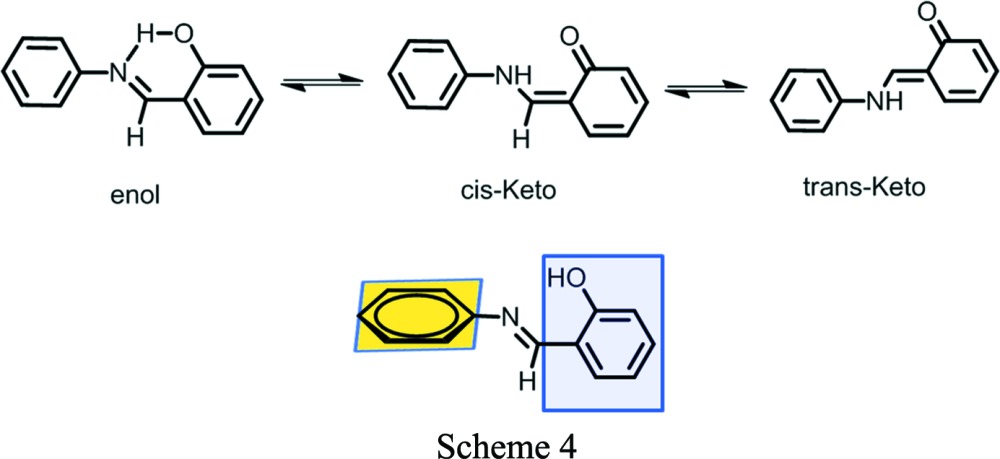


## 4. Conclusions   

In the present investigation we have explored the mechanical response of organic crystals towards thermal stress and the role of type II halogen bonding in tuning the properties of materials. The dichloro (Form I) derivative of the Schiff base shows excellent thermal effects, and a decrease in thermal effects was observed with the more polarizable (and less electronegative) iodine in the order Cl⋯Cl > Br⋯Cl > Br⋯Br > I⋯I. These effects are ascribed to anisotropy in the cell parameters and to the sudden release of accumulated strain during the phase transition. Our results demonstrate the importance of weak halogen-bonding interactions (Cl⋯Cl) of the type II variety in a family of thermally stable and highly reversible self-actuating materials.

## Supplementary Material

Crystal structure: contains datablock(s) Compound-A-Form-I, Compound-A-Form-II, Compound-A-Form-III, Compound-B-Form-I, Compound-B-Form-II, Compound-C-Form-I, Compound-C-Form-II, Compound-D, Compound-E. DOI: 10.1107/S2052252517014658/ed5012sup1.cif

Structure factors: contains datablock(s) Compound-A-Form-I. DOI: 10.1107/S2052252517014658/ed5012Compound-A-Form-Isup2.hkl

Structure factors: contains datablock(s) Compound-A-Form-II. DOI: 10.1107/S2052252517014658/ed5012Compound-A-Form-IIsup3.hkl

Structure factors: contains datablock(s) Compound-A-Form-III. DOI: 10.1107/S2052252517014658/ed5012Compound-A-Form-IIIsup4.hkl

Structure factors: contains datablock(s) Compound-B-Form-I. DOI: 10.1107/S2052252517014658/ed5012Compound-B-Form-Isup5.hkl

Structure factors: contains datablock(s) Compound-B-Form-II. DOI: 10.1107/S2052252517014658/ed5012Compound-B-Form-IIsup6.hkl

Structure factors: contains datablock(s) Compound-C-Form-I. DOI: 10.1107/S2052252517014658/ed5012Compound-C-Form-Isup7.hkl

Structure factors: contains datablock(s) Compound-C-Form-II. DOI: 10.1107/S2052252517014658/ed5012Compound-C-Form-IIsup8.hkl

Structure factors: contains datablock(s) Compound-D. DOI: 10.1107/S2052252517014658/ed5012Compound-Dsup9.hkl

Structure factors: contains datablock(s) Compound-E. DOI: 10.1107/S2052252517014658/ed5012Compound-Esup10.hkl

Supporting information file. DOI: 10.1107/S2052252517014658/ed5012Compound-Asup11.cml

Supporting information file. DOI: 10.1107/S2052252517014658/ed5012Compound-Bsup12.cml

Supporting information file. DOI: 10.1107/S2052252517014658/ed5012Compound-Csup13.cml

Supporting information file. DOI: 10.1107/S2052252517014658/ed5012Compound-Dsup14.cml

Additional tables and figures. DOI: 10.1107/S2052252517014658/ed5012sup15.pdf

CCDC references: 1579384, 1548278, 1548279, 1548280, 1548281, 1548282, 1548283, 1548284, 1548285

## Figures and Tables

**Table 1:** Selected halogen- and hydrogen-bond distances in all nine polymorphic structures

Compound	Polymorph	*X*⋯*X* (Å)	N—H⋯N (Å)
Compound-A	Forms I, II, III	3.435 (6), 3.395 (1), 3.448 (4)	2. 17 (1), 2.24 (2), 2.23 (1)
Compound-B	Forms I, II	3.587 (1), 3.415 (1)	2.13 (2)
Compound-C	Forms I, II	3.4962 (9), 3.50 (1)	2.16 (3), 2.22 (3)
Compound-D		3.8561 (5)	2.15 (5)
Compound-E			2.19 (1)

**Table 2:** Dihedral angles between the two rings C1–C6 and C9–C13/N3

Compound	Dihedral angle (°)
Compound-A Forms I, II and III	10.26 (8), 9.76 (1) and 10.95 (6)
Compound-B Form I	15.55 (3)
Compound-B Form II	41.36 (3)† and 4.47 (4)
Compound-C Forms I, II	12.05 (2), 10.54 (3)
Compound-D	19.72 (2)
Compound-E	5.34 (2)

† In Compound-B Form II, the dihedral angle of 41.36° is between the rings C14–C19 and C22–C26/N6.

**Table 3:** Unit-cell similarity index (Π)

Compound	Unit cell similarity index (Π)
Compounds-A and -B	0.0139
Compounds-B and -C	0.0020
Compounds-C and -D	0.0246
Compounds-D and -E	0.0735
Compounds-A and -D	0.0362
Compounds-A and -E	0.0344

**Table 4:** Strength of halogen bonds†

Compound	Halogen bond (*X*⋯*X*)	Stabilization energy (kcal mol^−1^)
Compound-A (Forms I and II)	Cl⋯Cl	−4.18, −5.31
Compound-B (Form I)	Br⋯Br	−4.39
Compound-C (Form I)	Cl⋯Br	−4.24
Compound-D	I⋯I	−4.70

† See Table S3 in the supporting information for full details.

**Table 5:** Cell parameters and phase transitions of the polymorphs

Transition	*T* (K) DSC	Form	*a* axis (Å)	*b* axis (Å)	*c* axis (Å)	Volume (Å^3^)
Compound-A	298–326	I†	8.0683 (4)	10.7464 (6)	15.6155 (8)	1353.78 (12)
I→III	330–332	III‡	8.6916 (6)	10.2641 (7)	15.4965 (10)	1382.46 (16)
		Difference (%)	7.72	−4.48	−0.76	2.11
Compound-B		I†	8.266 (2)	10.917 (2)	15.734 (3)	1412.7 (5)
I→II		II†	9.8084 (7)	10.7751 (8)	13.9516 (9)	1429.11 (17)
		Difference (%)	18.65	−1.29	−11.33	1.16
Compound-C	298–358	I†	8.2475 (9)	10.8035 (11)	15.7971 (15)	1399.6 (2)
I→II	360–366	II§	8.651 (2)	10.446 (3)	15.680 (4)	1413.3 (7)
		Difference (%)	4.89	−3.30	−0.74	0.97

† Unit cell measured at 298 K.

‡ Unit cell measured at 338 K.

§ Unit cell measured at 368 K.
