# Supplementary material for: Thermomechanical effect in molecular crystals: the role of halogen-bonding interactions
Source: IUCrJ. 2017 Oct 27;4(Pt 6):812–23. doi: 10.1107/S2052252517014658 (PMC5668866; doi:10.1107/S2052252517014658)
Supplement: Supplementary file 15 [file m-04-00812-sup15.pdf]

IUCrJ

Volume 5 (2018)

Supporting information for article:

Thermomechanical effect in molecular crystals: the role of halogen-bonding interactions

Sudhir Mittapalli, D. Sravanakumar Perumalla, Jagadeesh Babu Nanubolu and Ashwini Nangia

Table S1: Hydrogen/halogen bond geometry in crystal structures.

	**Interaction**	**D–H/Å**	**H···A/Å**	**D···A/Å**	**∠D–H···A/Å**	**Symmetry code**
A Form I	O2–H2A···N1	0.82	1.86	2.578(1)	145	Intramolecular
	N2–H2B···N3	0.86	2.17	2.9891(2)	156	-x,1/2+y,1/2-z
	C1–H1···Cl2	0.93	2.81	3.722(2)	166	x,3/2-y,-1/2+z
	C4–Cl2···Cl1			3.435(6)	155	Intermolecular
	C2–Cl1···Cl2			3.435(6)	96	Intermolecular
A Form II	O2–H2A···N1	0.84	1.88	2.617(2)	144	Intramolecular
	N2–H2B···N3	0.86	2.24	3.083(2)	159	-x,2-y,1/2+z
	C10–H10··· N3	0.95	2.59	3.511(2)	166	-x,2-y,1/2+z
	C11–H11···O1	0.95	2.40	3.341(2)	174	-1/2+x,2-y,z
	C13–H13··· Cl1	0.95	2.92	3.676(2)	136	x,1+y,-1+z
	C4–Cl2···Cl1			3.395(1)	151	Intermolecular
	C2–Cl1···Cl2			3.395(1)	100	Intermolecular
A Form III	O2–H2A···N1	0.82	1.88	2.591(11)	144	Intramolecular
	N2–H2B···N3	0.86	2.23	3.048(11)	159	2-x,-1/2+y,1/2-z
	C10–H10··· N3	0.93	2.56	3.466(14)	166	2-x,-1/2+y,1/2-z
	C11–H11··· O1	0.93	2.47	3.391(12)	174	x,1/2-y,1/2+z
	C4–Cl2···Cl1			3.448(4)	155.5	Intermolecular
	C2–Cl1···Cl2			3.448(4)	95	Intermolecular
B Form I	O2–H2A···N1	0.82	1.83	2.551(4)	146	Intramolecular
	N2–H2B···N3	0.86	2.13	2.920(5)	152	-x,-1/2+y,1/2-z
	C12–H12···O1	0.93	2.45	3.354(5)	165	x,1/2-y,1/2+z
	C4–Br2···Br1			3.587(1)	166	Intermolecular
	C2–Br1···Br2			3.587(1)	94	Intermolecular
	O2–H2A···N1	0.82	1.89	2.605(5)	143	Intramolecular
	N2–H2B···O3	0.80	2.15	2.933(6)	164	X,y,z
	O4–H4A···N4	0.82	1.88	2.598(6)	145	X,y,z
	N5–H5B···O1	0.73	2.11	2.836(6)	169	1-x,1-y,1-z

B Form II	C10–H10··· O3	0.93	2.51	3.417(7)	166	X,y,z
	C23–H23··· Br3	0.93	2.91	3.707(6)	145	1-x,1-y,-z
	C15–Br3···Br3			3.415(1)	143	Intermolecular
C Form I	O2–H2A···N1	0.82	1.85	2.565(3)	145	Intramolecular
	N2–H2B···N3	0.86	2.16	2.966(3)	156	2-x,1/2+y,1/2-z
	C11–H11···O1	0.93	2.46	3.391(3)	173	x,1/2-y,-1/2+z
	C4–Br1···Cl1			3.4962(9)	167	Intermolecular
	C2–Cl1···Br1			3.4962(9)	98	Intermolecular
C Form II	O2–H2A···N1	0.82	1.86	2.568(4)	145	Intramolecular
	N2–H2B···N3	0.86	2.22	3.028(9)	156	-x,1/2+y,1/2-z
	C11–H11···O1	0.93	2.50	3.427 (4)	177	x,3/2-y,1/2+z
	C4–Br1···Cl1			3.50(1)	163	Intermolecular
	C2–Cl1···Br1			3.50(1)	98	Intermolecular
Compound-D	O2–H2A···N1	0.84	1.83	2.564(2)	145	Intramolecular
	N2–H2B···N3	0.88	2.15	2.920(2)	146	-x,-1/2+y,1/2-z
	C4–I2···I1			3.8561(5)	170	Intermolecular
	C2–I1···I2			3.8561(5)	92	Intermolecular
Compound-E	O2–H2A···N1	0.82	1.90	2.6062(7)	144	Intramolecular
	N2–H2B···N3	0.86	2.19	3.0130(8)	162	1-x,-1/2+y,3/2-z

Table S2: Crystallographic information.

	**Compound-A Form I**	**Compound-A Form II**	**Compound-A Form III**	**Compound-B Form I**	**Compound-B Form II**
Empirical Formula	$C_{13}H_9Cl_2N_3O_2$	$C_{13}H_9Cl_2N_3O_2$	$C_{13}H_9Cl_2N_3O_2$	$C_{13}H_9Br_2N_3O_2$	$C_{13}H_9Br_2N_3O_2$
Formula weight	310.13	310.13	310.13	399.05	399.05
Crystal system	Monoclinic	Orthorhombic	Monoclinic	Monoclinic	Triclinic
Space group	$P2_1/c$	$Pca2_1$	$P2_1/c$	$P2_1/c$	P-1
T (K)	298	298	338	298	298

a (Å)	8.0683(4)	15.441(3)	8.6916(6)	8.266(2)	9.8084(7)
b (Å)	10.7464(6)	8.3179(16)	10.2641(7)	10.917(2)	10.7751(8)
c (Å)	15.6155(8)	10.6014(18)	15.4965(10)	15.734(3)	13.9516(9)
α (deg)	90	90	90	90	103.219(3)
β (deg)	90.890(2)	90	90.186(2)	95.82(3)	94.907(3)
γ (deg)	90	90	90	90	91.018(3)
V (Å^3)	1353.78(12)	1361.6(5)	1382.46(16)	1412.7(5)	1429.11(17)
D_{calcd} (g cm^{-3})	1.522	1.513	1.490	1.876	1.855
μ (mm^{-1})	0.483	0.480	0.473	5.742	5.676
θ range	2.30 to 27.57	2.44 to 25.03	2.34 to 25.07	3.10 to 28.05	2.45 to 24.79
Z/Z$'$	4/1	4/1	4/1	4/1	4/2
Range *h*	–10 to 10	–18 to 18	–10 to 9	–10 to 10	–11 to 11
Range *k*	–13 to 13	–9 to 9	–12 to 12	–14 to 14	–12 to 12
Range *l*	–20 to 20	–12 to 12	–18 to 18	–20 to 20	–16 to 16
Reflections collected	35148	8992	12375	18639	28553
Total reflections	3124	2379	2046	3402	4894
Observed reflections	2690	2279	1677	2697	4061
R_1 [$I > 2\sigma(I)$]	0.032	0.024	0.0811	0.0470	0.0478
$wR2$ (all)	0.0916	0.0626	0.2943	0.1028	0.1180
Goodness-of-fit	1.034	1.096	1.535	1.053	1.042
X-ray diffractometer	Bruker D8 Quest	Bruker D8 Quest	Bruker D8 Venture	Bruker D8 Quest	Bruker D8 Quest
CCDC Numbers	824932	1548278	1548279	1548280	1548281

	**Compound-C Form I**	**Compound-C Form II**	**Compound-D**	**Compound-E**
Empirical Formula	$C_{13}H_9BrClN_3O_2$	$C_{13}H_9BrClN_3O_2$	$C_{13}H_9I_2N_3O_2$	$C_{13}H_9F_2N_3O_2$
Formula weight	354.59	354.59	493.03	277.23
Crystal system	Monoclinic	Monoclinic	Monoclinic	Monoclinic
Space group	$P2_1/c$	$P2_1/c$	$P2_1/c$	$P2_1/c$
T (K)	298	368	298	298
a (Å)	8.2475(9)	8.651(2)	8.2946(5)	7.897(2)
b (Å)	10.8035(11)	10.446(3)	11.2673(6)	10.396(2)
c (Å)	15.7971(15)	15.680(4)	16.2177(10)	14.989(3)
α (deg)	90	90	90	90.00(3)
β (deg)	96.098(3)	94.116(11)	97.993(2)	92.27(3)
γ (deg)	90	90	90	90.00(3)
V (Å^3)	1399.6(2)	1413.3(7)	1500.95(15)	1229.6(5)
D_{calcd} (g cm^{-3})	1.683	1.667	2.182	1.498
μ (mm^{-1})	3.131	3.101	4.194	0.124
θ range	2.28 to 25.00	2.34 to 28.88	2.208 to 25.021	2.581 to 26.005
Z/Z$'$	4/1	4/1	4/1	4/1
Range h	–9 to 9	–11 to 11	–9 to 9	–9 to 9
Range k	–12 to 12	–13 to 14	–13 to 13	–12 to 12
Range l	–18 to 18	–21 to 21	–19 to 19	–18 to 18
Reflections collected	10084	23004	9640	30614
Total reflections	2457	3582	2587	2336

Observed reflections	2198	2117	2357	1807
R_1 [I> 2 σ (I)]	0.0354	0.0878	0.0272	0.0457
wR_2 (all)	0.0990	0.2015	0.0698	0.1229
Goodness-of-fit	1.077	1.143	1.111	1.096
X-ray diffractometer	Bruker D8 Quest	Bruker D8 Venture	Bruker D8 Quest	Bruker D8 Quest
CCDC Numbers	1548282	1548283	1548284	1548285

Table S3: Stabilization energies of halogen bonds.

**Name of the Compound**	**Moiety**	**Stabilization Energy (kCal/mol)**
**Compound-A Form I**	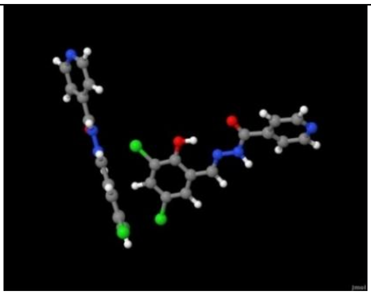	**-4.18**
**Compound-A Form II**	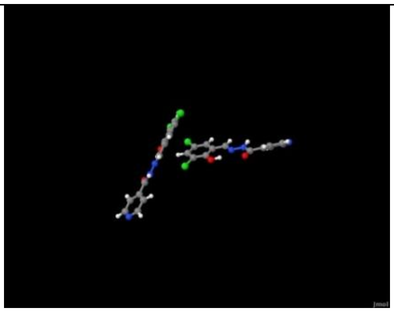	**-5.31**
**Compound-B Form I**	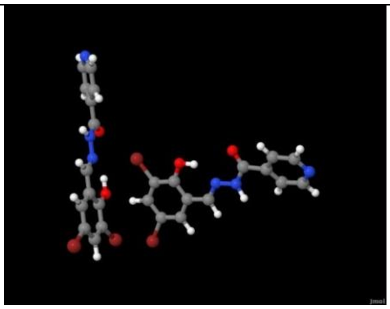	**-4.39**

**Compound-C Form I**	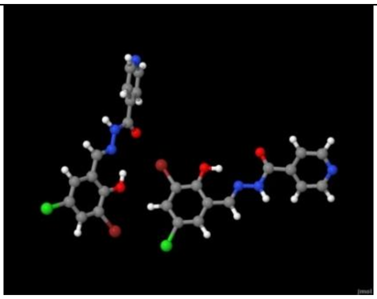	**-4.24**
**Compound-D**	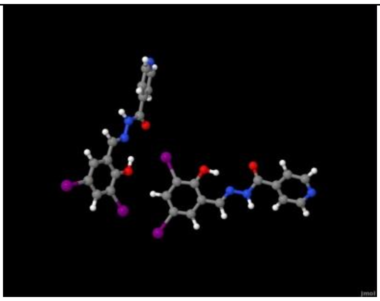	**-4.70**

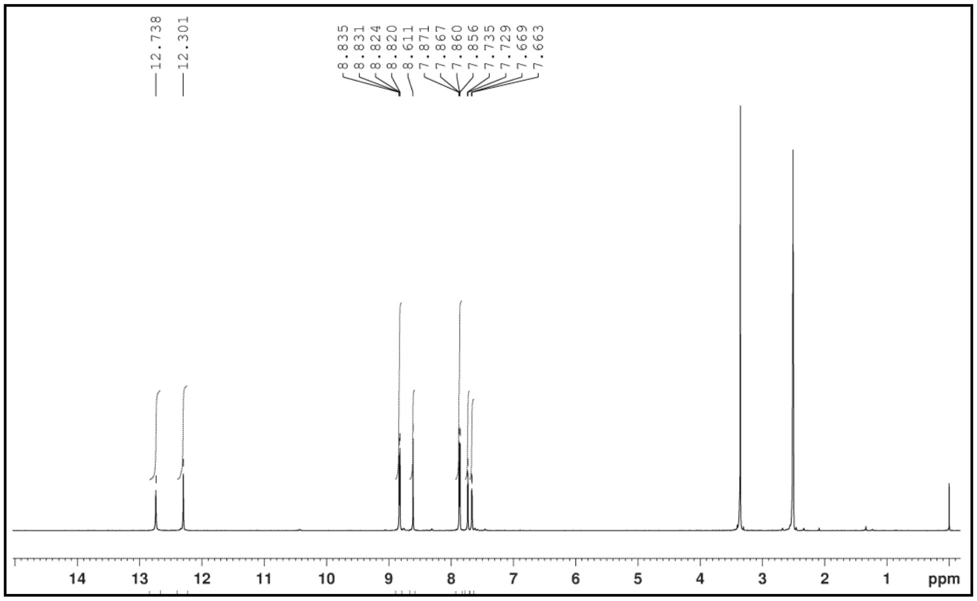

Figure S1a: Compound-A ^{1}H-NMR.

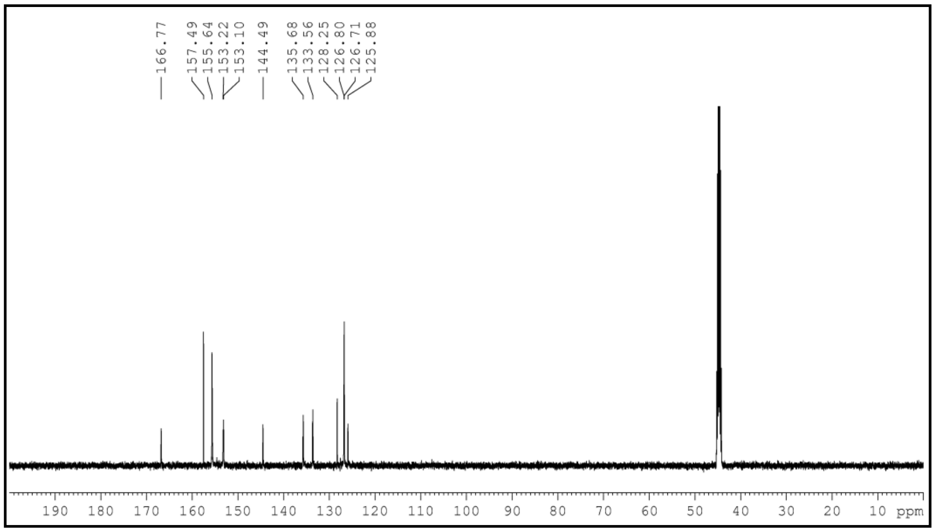

Figure S1b: Compound-A ^{13}C-NMR.

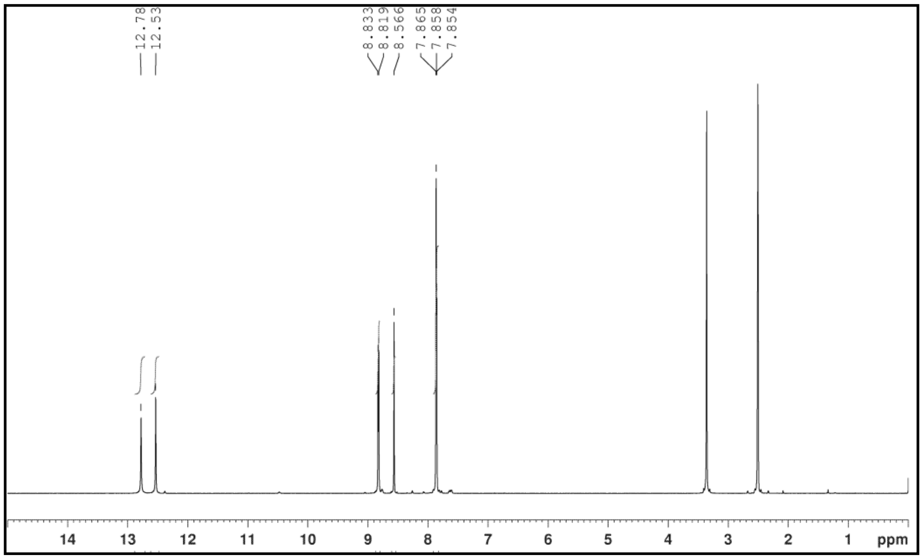

Figure S1c: Compound-B ^{1}H-NMR.

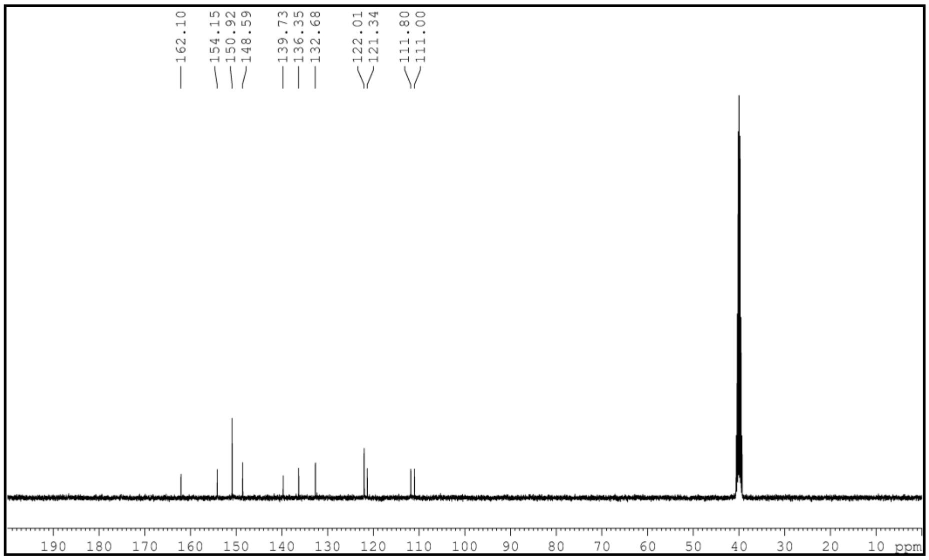

Figure S1d: Compound-B ^{13}C-NMR.

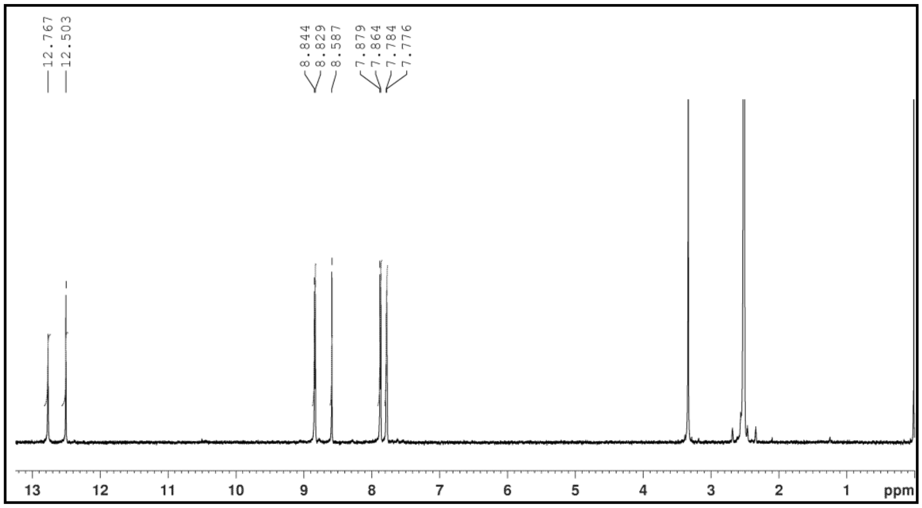

Figure S1e: Compound-C ^{1}H-NMR.

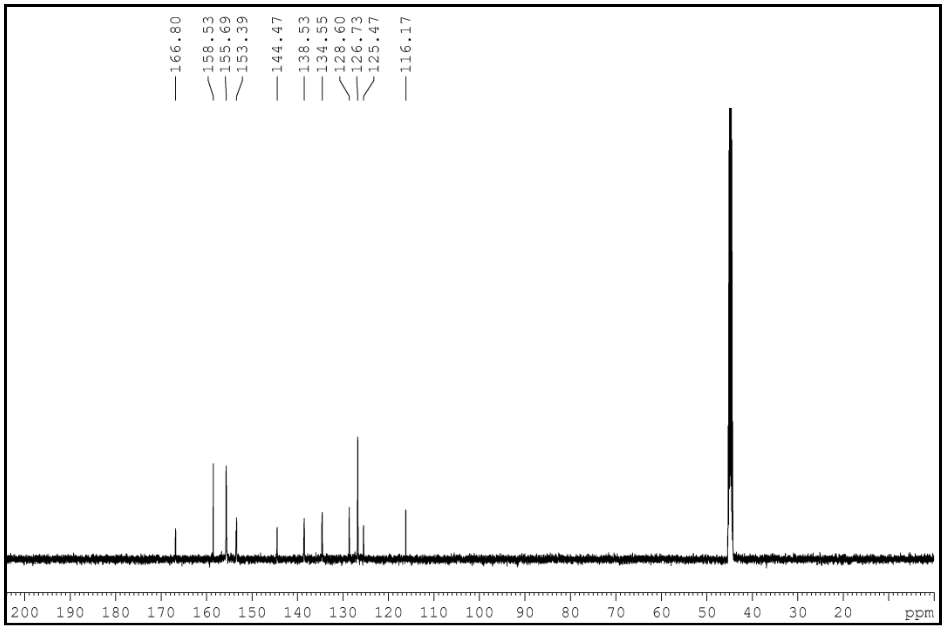

Figure S1f: Compound-C ^{13}C-NMR.

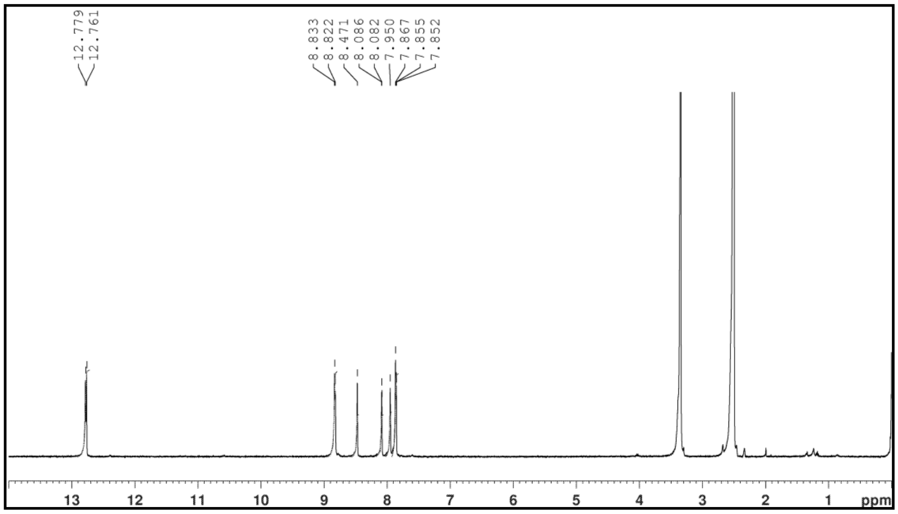

Figure S1g: Compound-D ^{1}H-NMR.

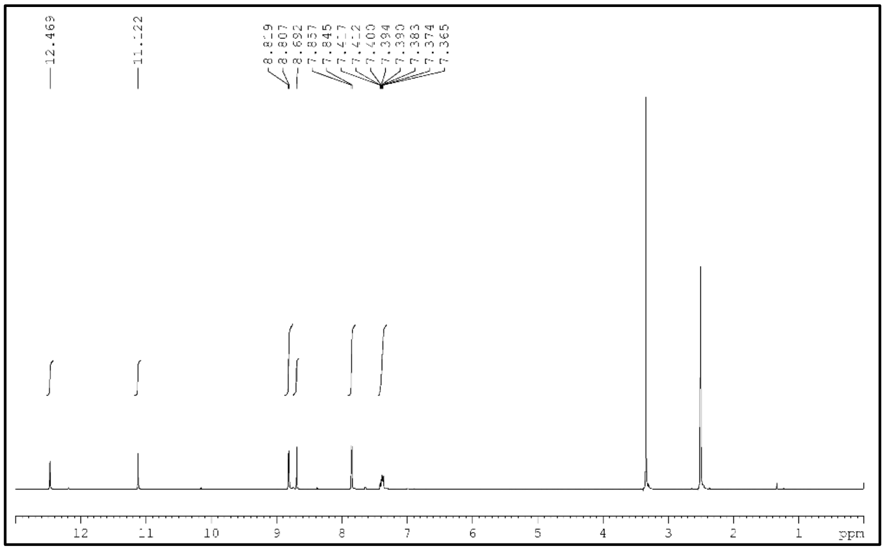

Figure S1h: Compound-E ^{1}H-NMR.

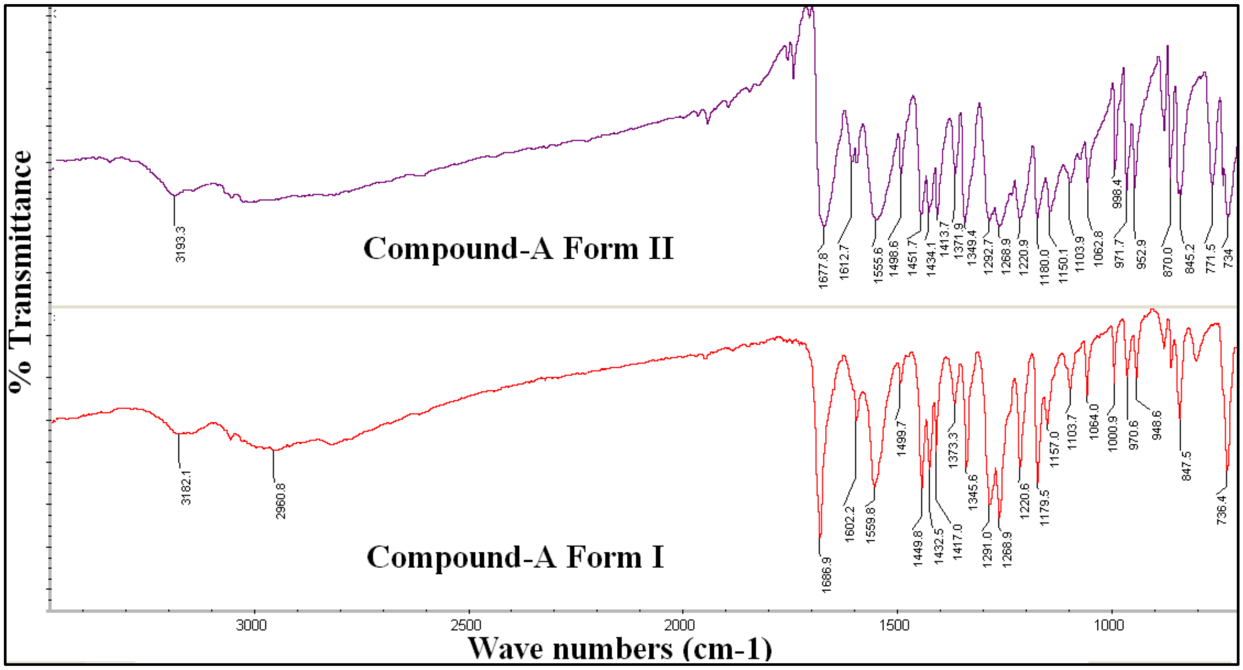

Figure S2a: FT-IR comparison for Form I and Form II (Compound-A)

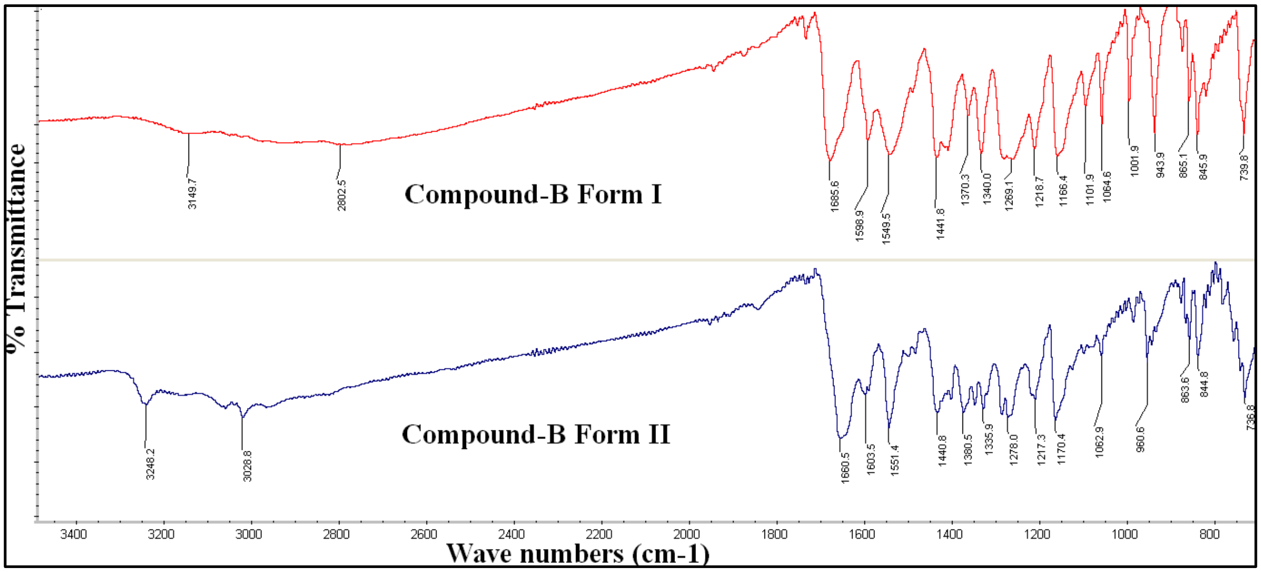

Figure S2b: FT-IR comparison for Form I and Form II (Compound-B)

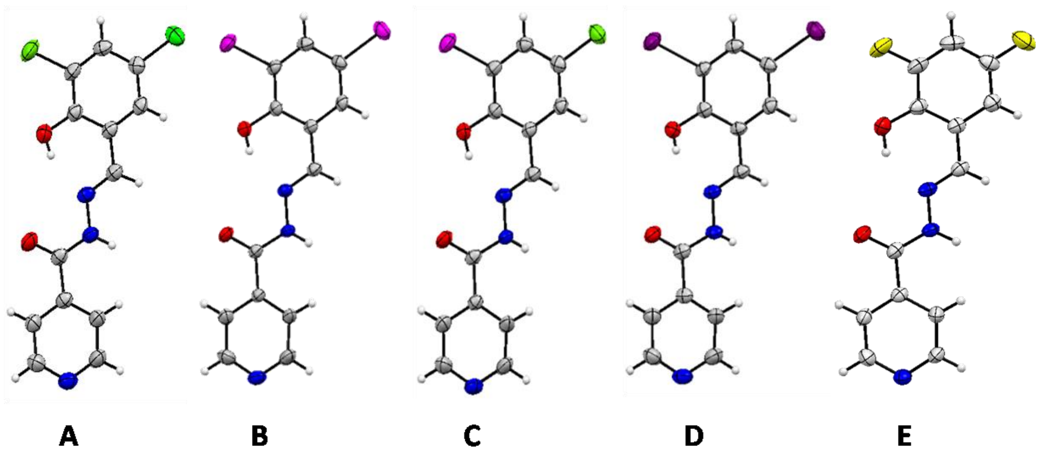

A= (E)-N'-(3,5-dichloro-2-hydroxybenzylidene)isonicotinohydrazide

B= (E)-N'-(3,5-dibromo-2-hydroxybenzylidene)isonicotinohydrazide

C= (E)-N'-(3-bromo-5-chloro-2-hydroxybenzylidene)isonicotinohydrazide

D= (E)-N'-(3,5-diiodo-2-hydroxybenzylidene)isonicotinohydrazide

E= (E)-N'-(3,5-difluoro-2-hydroxybenzylidene)isonicotinohydrazide

Figure S3: ORTEP diagram of salinazids structures.

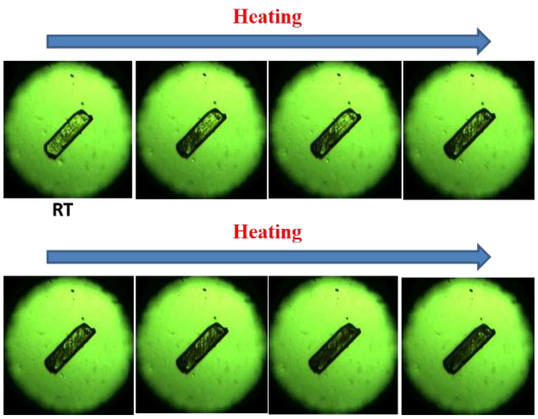

Figure S4: Hot stage microscope images during heating of Compound-B thermosalient crystals.

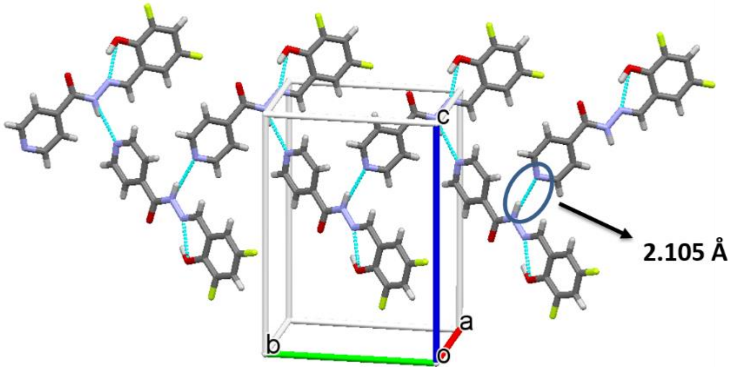

Figure S5: Chain extending through N-H…N H bond interactions (Compound-E).

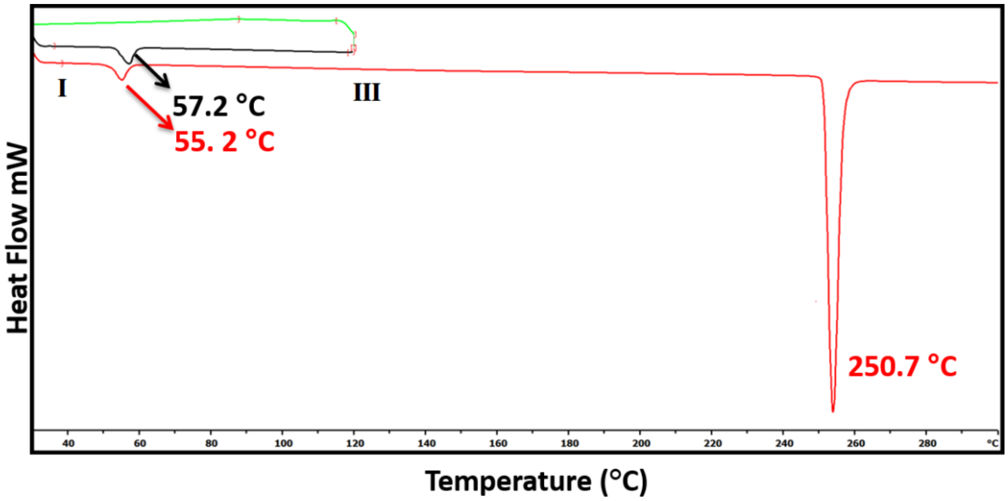

Figure S6a: DSC thermogram of Compound-A Form I.

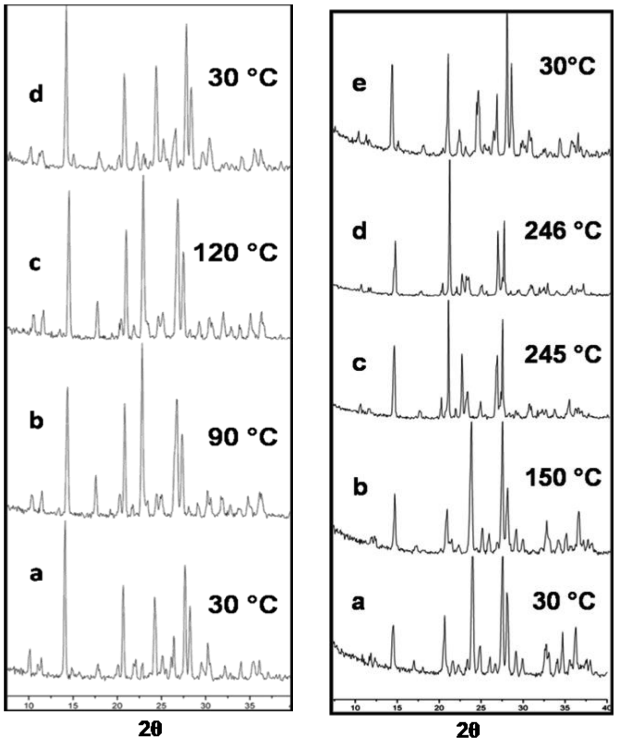

a b

Figure S6b: VT-PXRD for Compound-A Form I (a) and II (b).

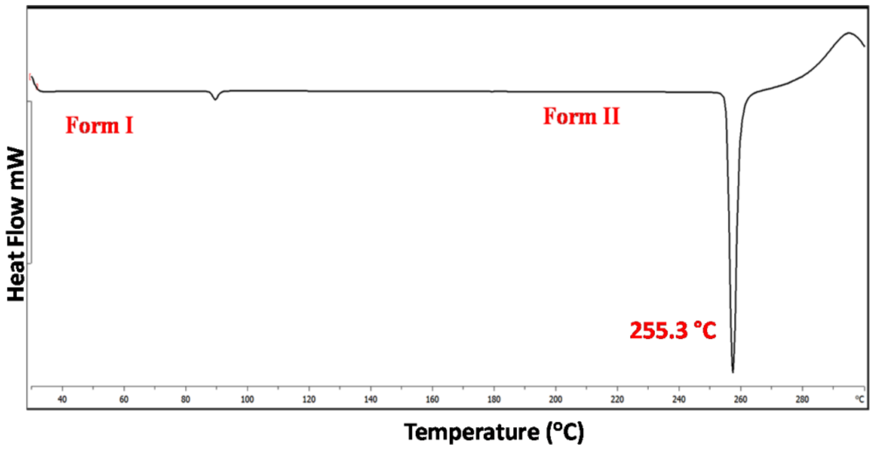

Figure S7a: DSC thermogram of Compound-C.

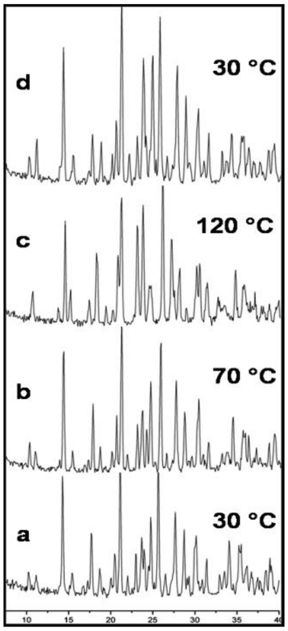

Figure S7b: VT-PXRD for Compound-C Form I (label a) which converts to Form II at 120 °C (label c).

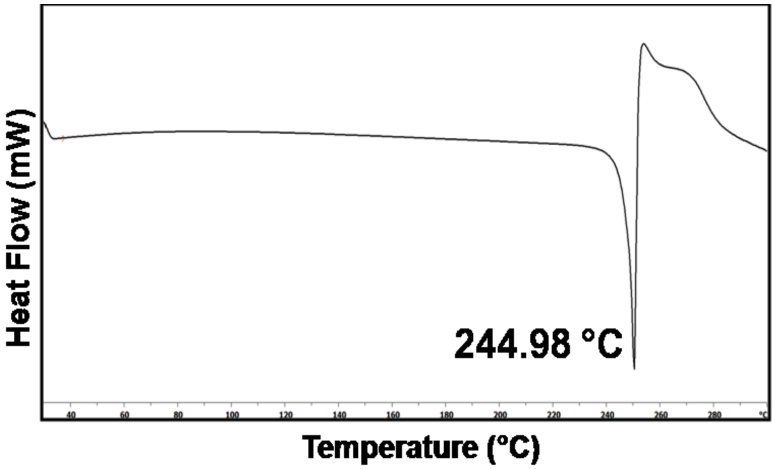

Figure S8: DSC thermogram of Compound-D.

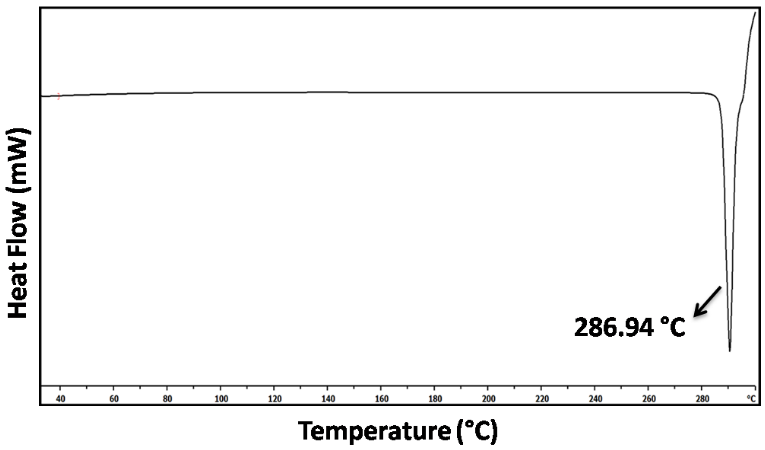

Figure S9: DSC thermogram of Compound-E.

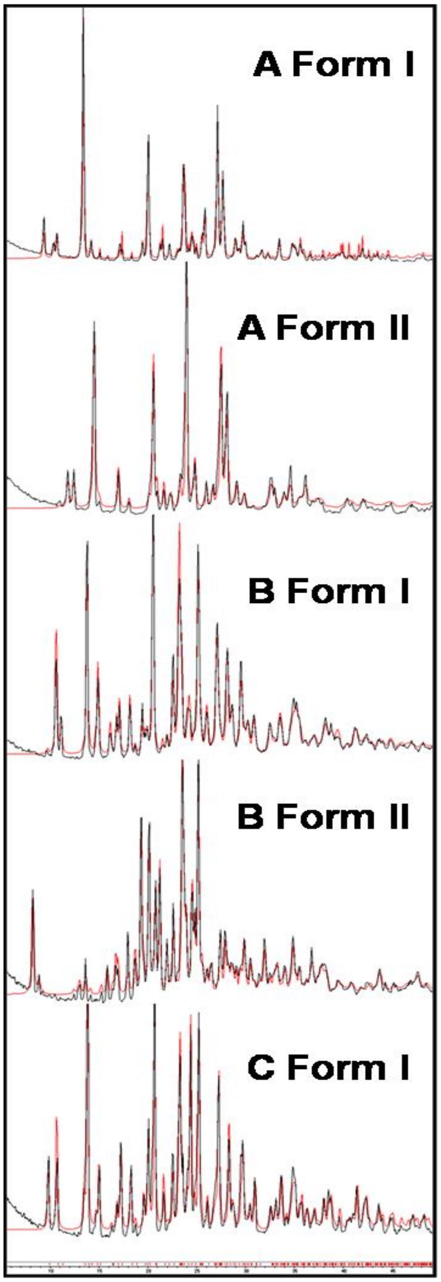

Figure S10: Overlay of experimental PXRD pattern with calculated lines from the X-ray structure.

XPac Analysis:

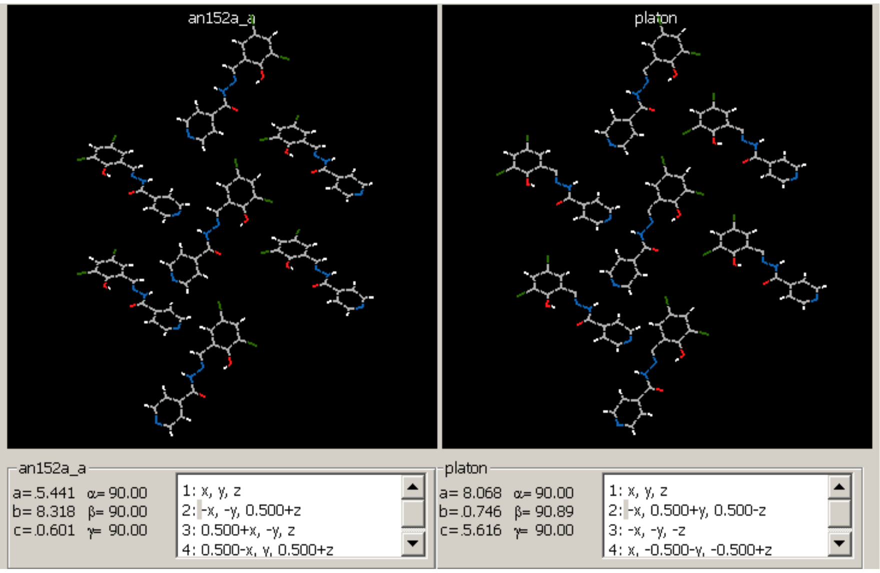

(a)

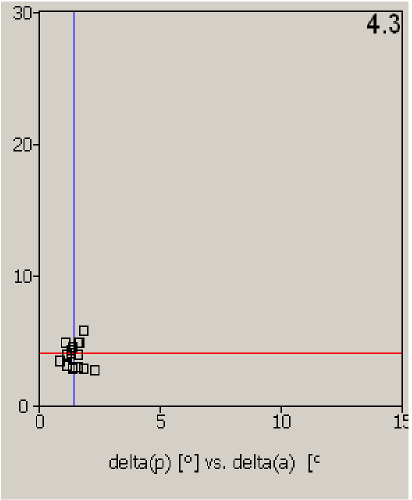

(b)

Figure S11a: (a) XPac analysis of **c**ompound-A Form I and II. (b) The interplanar angular deviation (δp, x-axis) vs. angular deviation (δa, y-axis) plot (in °) indicates a dissimilarity index of 4.3 due to the 3D isostructurality.

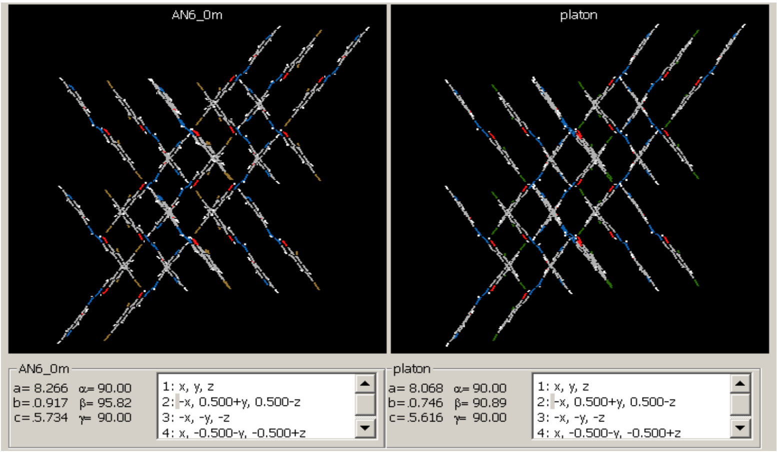

(a)

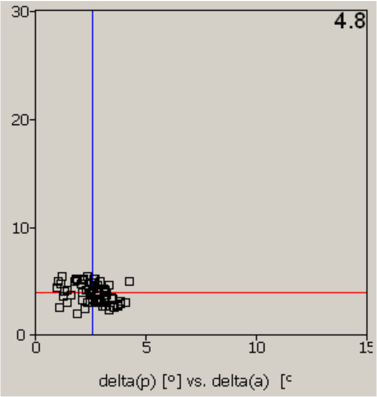

(b)

Figure S11b: (a) XPac analysis of compound-A and B. (b) The interplanar angular deviation (δp, x-axis) vs. angular deviation (δa, y-axis) plot (in °) indicates a dissimilarity index of 4.8 due to the 3D isostructurality.

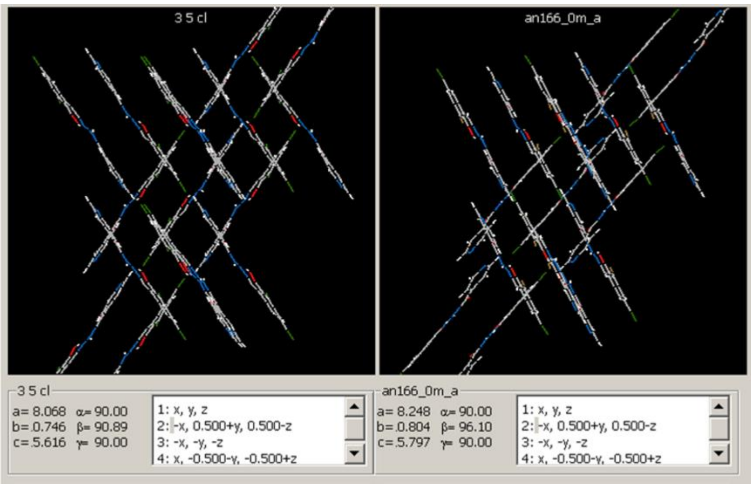

(a)

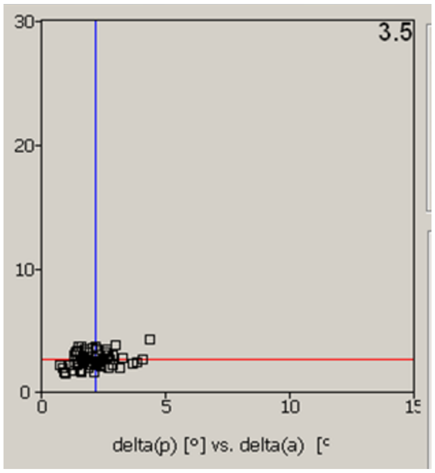

(b)

Figure S11c: (a) XPac analysis of compound-A and C. (b) The interplanar angular deviation (δp, x-axis) vs. angular deviation (δa, y-axis) plot (in °) indicates a dissimilarity index of 3.5 due to the 3D isostructurality.

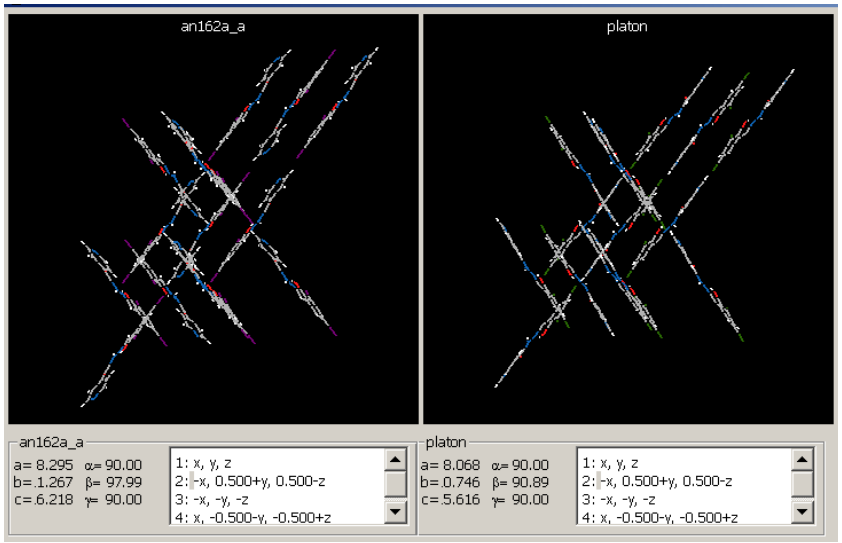

(a)

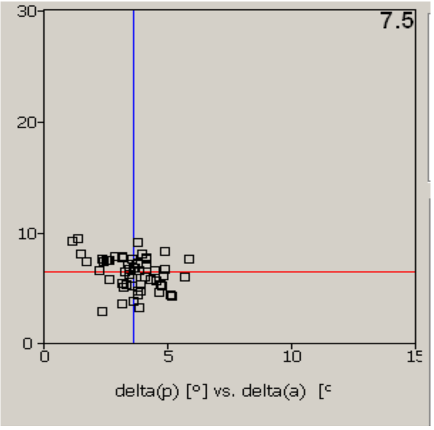

(b)

Figure S11d: (a) XPac analysis of compound-A and D. (b) The interplanar angular deviation (δp, x-axis) vs. angular deviation (δa, y-axis) plot (in °) indicates a dissimilarity index of 7.5 due to the 3D isostructurality.

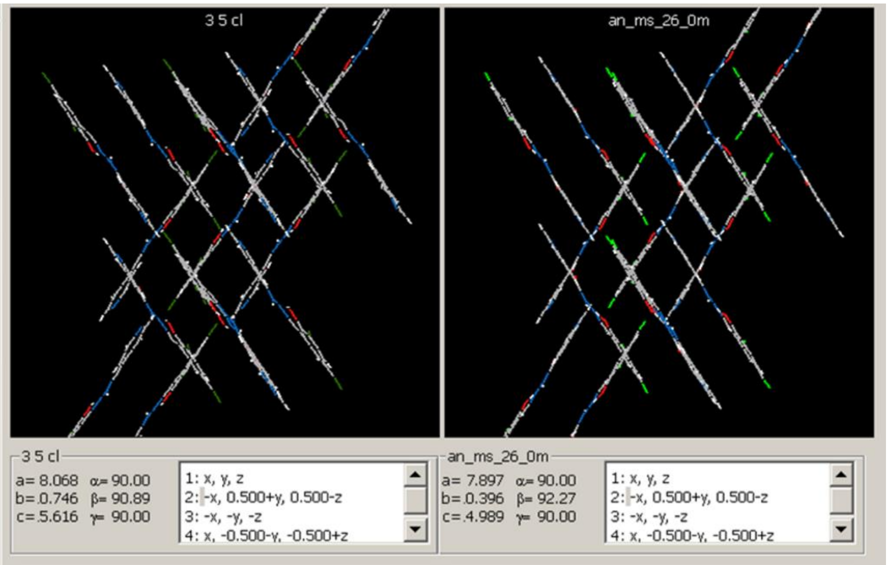

(a)

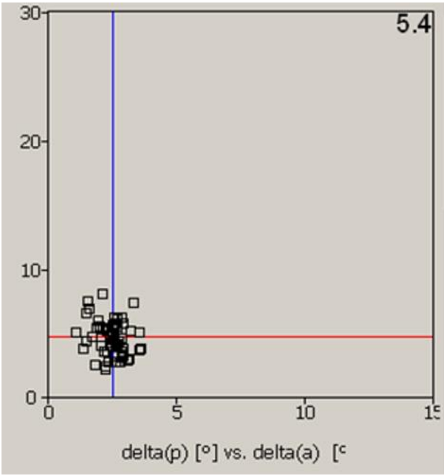

(b)

Figure S11e: (a) XPac analysis of compound-A and E. (b) The interplanar angular deviation (δp, x-axis) vs. angular deviation (δa, y-axis) plot (in °) indicates a dissimilarity index of 5.4 due to the 3D isostructurality.

Hirshfeld Surface Analysis:

Compound-A Form I:

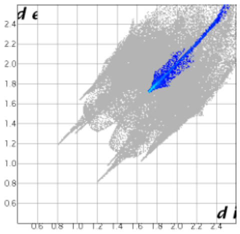
Cl…Cl 3.0%

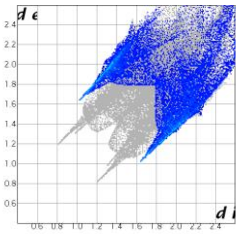
Cl…H 21.9%

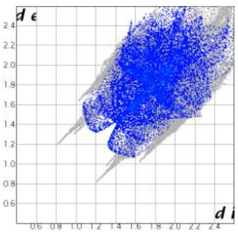
H…H 19.1%

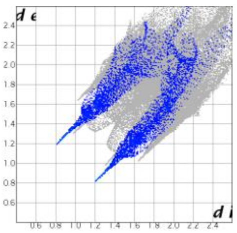
N…H 7.6%

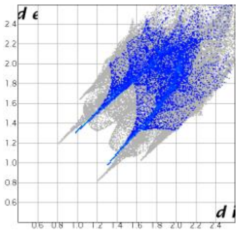
O…H 13.2%

Compound-A Form II:

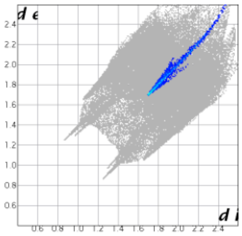
Cl…Cl 2.8%

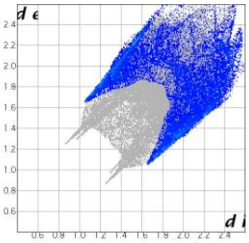
Cl…H 21.7%

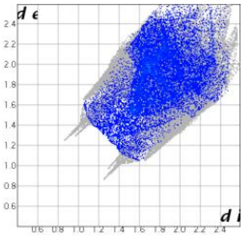
H…H 18.5%

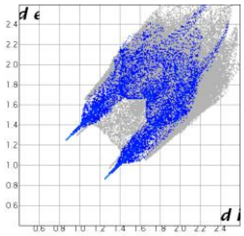
N…H 10.2%

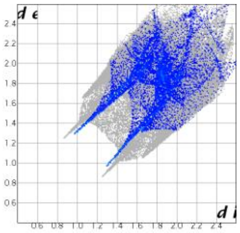
O…H 12.6%

Compound-A Form III:

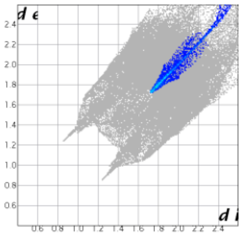
Cl…Cl 3.9%

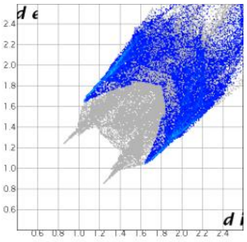
Cl…H 21.5%

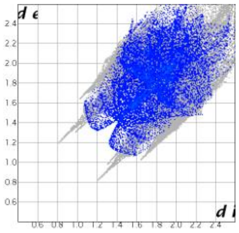
H…H 19.9%

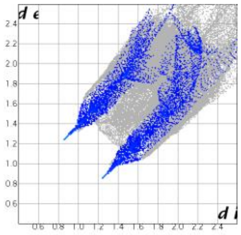
N…H 8.4%

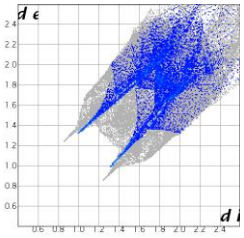
O…H 13.6%

Compound-B Form I:

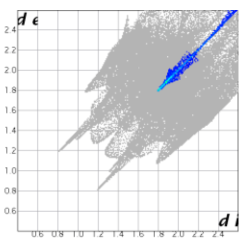 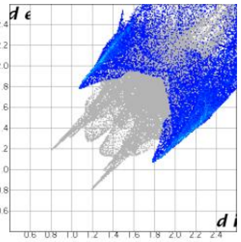 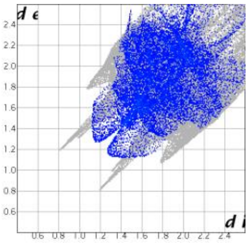 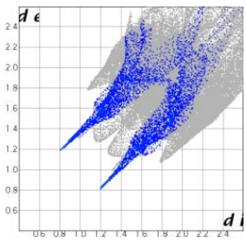 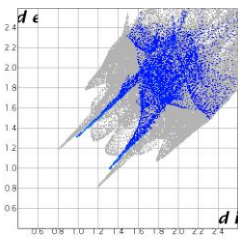

Br…Br 3.2% **Br…H 23.2%** **H…H 18.6%** **N…H 7.4%** **O…H 9.8%**

Compound-B Form II:

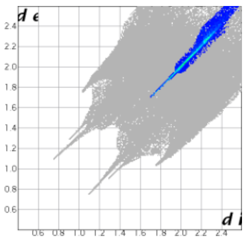 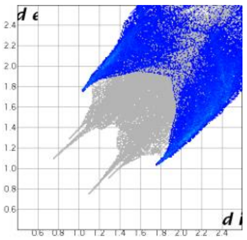 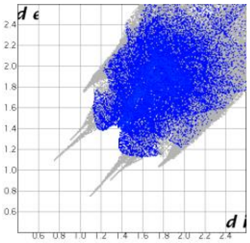 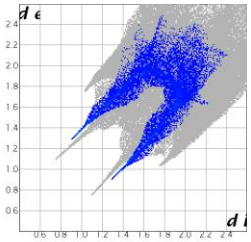 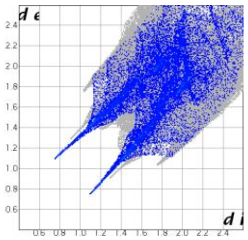

Br…Br 5.9% **Br…H 25.8%** **H…H 15.8%** **N…H 8.1%** **O…H 11.5%**

Compound-C Form I:

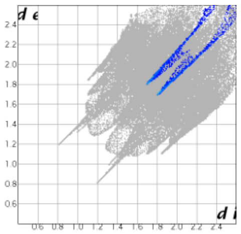 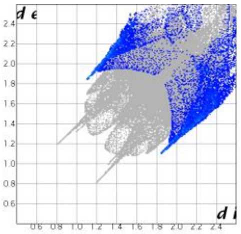 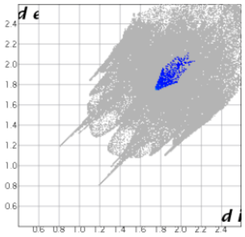 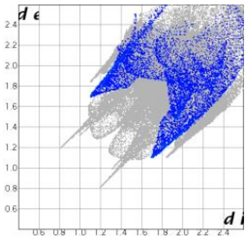 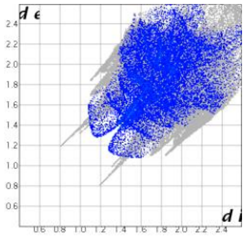

Br…Cl 2.6% **Br…H 12.9%** **Cl…Cl 0.7%** **Cl…H 9.7%** **H…H 20.3%**

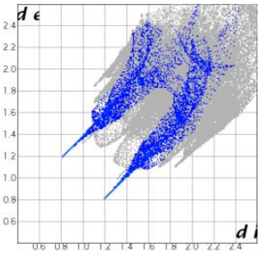 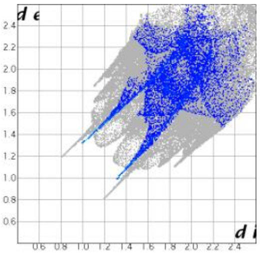

N…H 7.7% **O…H 10.6%**

Compound-C Form II:

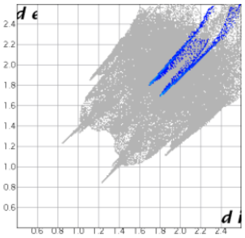 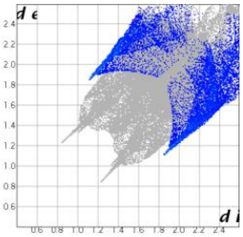 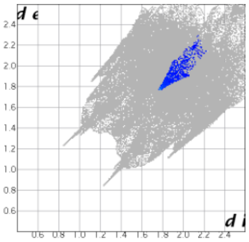 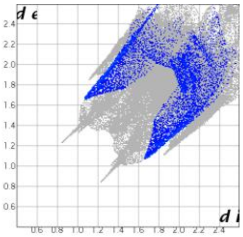 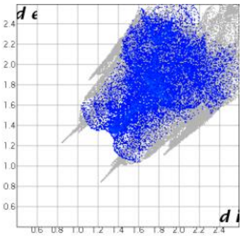

Br…Cl 2.8% **Br…H 13.2%** **Cl…Cl 1.1%** **Cl…H 8.4%** **H…H 20.5%**

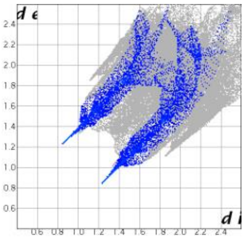 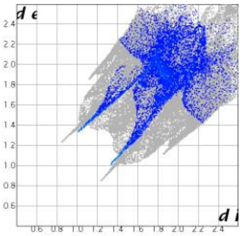

N…H 8.4% **O…H 12.2%**

Compound-D:

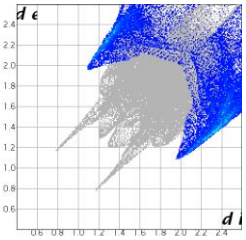 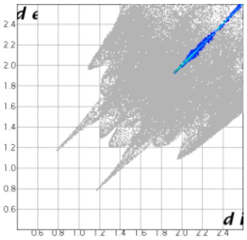 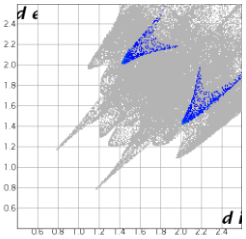 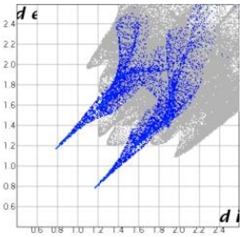 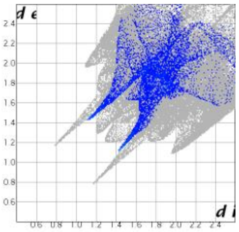

I…H 21.3% **I…I 3.4%** **I…O 1.5%** **N…H 7.1%** **O…H 7.9%**

Figure S12: Hirshfeld Surface analysis for Compound A-D.

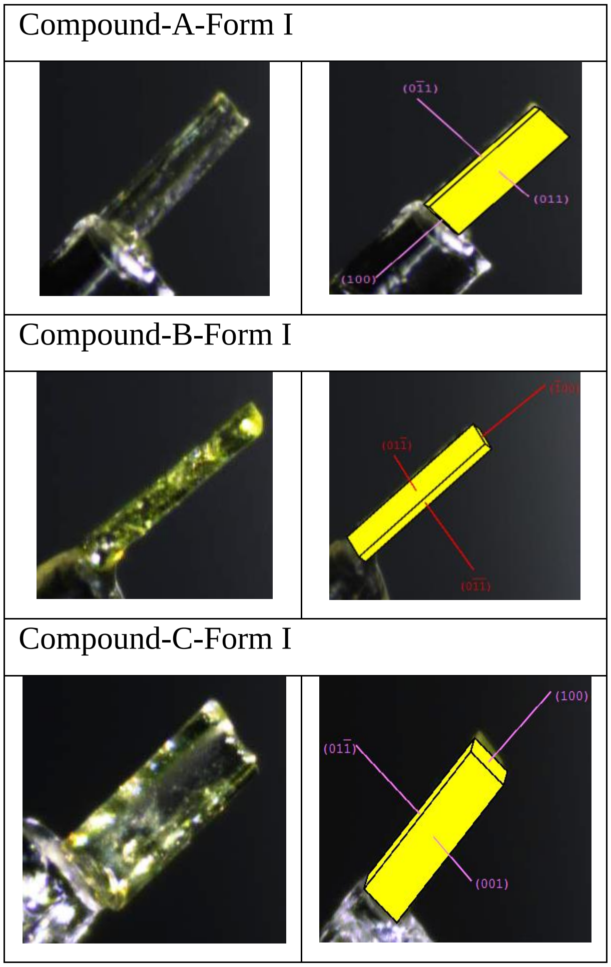

Figure S13: Morphology and faces of crystals.
